# DNA methylation-based subtype prediction for pediatric acute lymphoblastic leukemia

**DOI:** 10.1186/s13148-014-0039-z

**Published:** 2015-02-17

**Authors:** Jessica Nordlund, Christofer L Bäcklin, Vasilios Zachariadis, Lucia Cavelier, Johan Dahlberg, Ingegerd Öfverholm, Gisela Barbany, Ann Nordgren, Elin Övernäs, Jonas Abrahamsson, Trond Flaegstad, Mats M Heyman, Ólafur G Jónsson, Jukka Kanerva, Rolf Larsson, Josefine Palle, Kjeld Schmiegelow, Mats G Gustafsson, Gudmar Lönnerholm, Erik Forestier, Ann-Christine Syvänen

**Affiliations:** Department of Medical Sciences, Molecular Medicine and Science for Life Laboratory, Uppsala University, Box 1432, BMC, SE-751 44 Uppsala, Sweden; Department of Medical Sciences, Cancer Pharmacology and Computational Medicine, Uppsala University, Uppsala University Hospital, Entrance 40, SE-751 85 Uppsala, Sweden; Department of Molecular Medicine and Surgery, Karolinska Institutet, SE-171 76 Stockholm, Sweden; Department of Immunology, Genetics and Pathology, Uppsala University, Rudbecklaboratoriet, SE-751 85 Uppsala, Sweden; Department of Pediatrics, Queen Silvia Children’s Hospital, Rondvägen 10, SE-416 85 Gothenburg, Sweden; Department of Pediatrics, Tromsø University and University Hospital, Sykehusveien 38, N-9038 Tromsø, Norway; Childhood Cancer Research Unit, Karolinska Institutet, Astrid Lindgren Children’s Hospital, Karolinska University Hospital, Q6:05, SE-171 76, Stockholm, Sweden; Pediatric Hematology-Oncology, Children’s Hospital, Barnaspitali Hringsins, Landspitali University Hospital, Norðurmýri, 101, Reykjavik, Iceland; Division of Hematology-Oncology and Stem Cell Transplantation, Children’s Hospital, Helsinki University Central Hospital and University of Helsinki, Box 281, FIN-00029 Helsinki, Finland; Department of Women’s and Children’s Health, Pediatric Oncology, Uppsala University, Uppsala University Hospital, Entrance 95, SE-751 85 Uppsala, Sweden; Pediatrics and Adolescent Medicine, Rigshospitalet, and the Medical Faculty, Institute of Clinical Medicine, University of Copenhagen, Blegdamsvej 9, DK-2100 Copenhagen, Denmark; Department of Medical Biosciences, University of Umeå, SE-901 85 Umeå, Sweden

**Keywords:** DNA methylation, Pediatric acute lymphoblastic leukemia, CpG site, Subtyping, Cytogenetics, RNA-seq, 450 k array, epigenetics

## Abstract

**Background:**

We present a method that utilizes DNA methylation profiling for prediction of the cytogenetic subtypes of acute lymphoblastic leukemia (ALL) cells from pediatric ALL patients. The primary aim of our study was to improve risk stratification of ALL patients into treatment groups using DNA methylation as a complement to current diagnostic methods. A secondary aim was to gain insight into the functional role of DNA methylation in ALL.

**Results:**

We used the methylation status of ~450,000 CpG sites in 546 well-characterized patients with T-ALL or seven recurrent B-cell precursor ALL subtypes to design and validate sensitive and accurate DNA methylation classifiers. After repeated cross-validation, a final classifier was derived that consisted of only 246 CpG sites. The mean sensitivity and specificity of the classifier across the known subtypes was 0.90 and 0.99, respectively. We then used DNA methylation classification to screen for subtype membership of 210 patients with undefined karyotype (normal or no result) or non-recurrent cytogenetic aberrations (‘other’ subtype). Nearly half (*n* = 106) of the patients lacking cytogenetic subgrouping displayed highly similar methylation profiles as the patients in the known recurrent groups. We verified the subtype of 20% of the newly classified patients by examination of diagnostic karyotypes, array-based copy number analysis, and detection of fusion genes by quantitative polymerase chain reaction (PCR) and RNA-sequencing (RNA-seq). Using RNA-seq data from ALL patients where cytogenetic subtype and DNA methylation classification did not agree, we discovered several novel fusion genes involving *ETV6*, *RUNX1*, and *PAX5*.

**Conclusions:**

Our findings indicate that DNA methylation profiling contributes to the clarification of the heterogeneity in cytogenetically undefined ALL patient groups and could be implemented as a complementary method for diagnosis of ALL. The results of our study provide clues to the origin and development of leukemic transformation. The methylation status of the CpG sites constituting the classifiers also highlight relevant biological characteristics in otherwise unclassified ALL patients.

**Electronic supplementary material:**

The online version of this article (doi:10.1186/s13148-014-0039-z) contains supplementary material, which is available to authorized users.

## Background

The genetic subtypes of pediatric acute lymphoblastic leukemia (ALL) are characterized by large-scale chromosomal aberrations, such as aneuploidies and translocations [1-3]. Karyotyping, fluorescent *in situ* hybridization (FISH), reverse transcriptase polymerase chain reaction (RT-PCR), and array-based methods for copy number analysis are routinely used to detect high hyperdiploidy (HeH, 51-67 chromosomes), the translocations t(9;22)(q34;q11)[*BCR/ABL1*], t(12;21)(p13;q22)[*ETV6/RUNX1*], t(1;19)(q23;p13.3)[*TCF3/PBX1*], 11q23/*MLL*-rearrangement, dic(9;20)(p13.2;q11.2), and intrachromosomal amplification of chromosome 21 iAMP21[*RUNX1* X >3], which are recurrent in patients with ALL. Therapy intensity for ALL patients is determined by risk assessment based on presenting features, such as white blood cell count, B- or T-lineage, genetic aberrations, and minimal residual disease after induction treatment [4,5]. The accuracy of detecting chromosomal abnormalities by karyotyping, FISH, and PCR is generally high; however, these methods do not allow detection of all the aberrations that may occur [6]. Moreover, 15% of ALL patients harbor complex, non-recurrent genomic aberrations and would benefit from improved diagnostic subtyping to identify potential high-risk aberrations.

Methylation of cytosine (5mC) residues in CpG dinucleotides is an epigenetic modification that plays a pivotal role in the establishment of cellular identity by influencing gene expression [7,8]. There are approximately 28 million CpG sites in the human genome that are targets for DNA methylation. The pathogenesis and phenotypic characteristics of leukemic cells are partially explained by specific and genome-wide alterations in DNA methylation [9-17]. We and others have previously observed a strong correlation between cytogenetic subtype and DNA methylation in ALL, which indicates that DNA methylation profiling may serve as a proxy for cytogenetic analysis [11,12,14,18].

Herein, we used our previously published 450 k DNA methylation profiling dataset [14] from >500 primary ALL samples comprising eight known recurrent subtypes of ALL to design and evaluate DNA methylation classifiers for subtype prediction. Using extensive cross-validation and methylation-based subtyping in an independently derived set of ALL patient samples, we show that DNA methylation classification is a highly sensitive and specific method for ALL subtyping. Finally, we aimed to ascertain subtype membership of 210 ALL patients where no subtype information is available and verified the DNA methylation-based subtype predictions with copy number analysis and detection of fusion genes. The classifier and code required for DNA methylation classification can be freely downloaded at https://github.com/Molmed/Nordlund-ALL-subtyping.

## Results

### Prediction of ALL subtypes using DNA methylation classifiers

**Figure 1:**
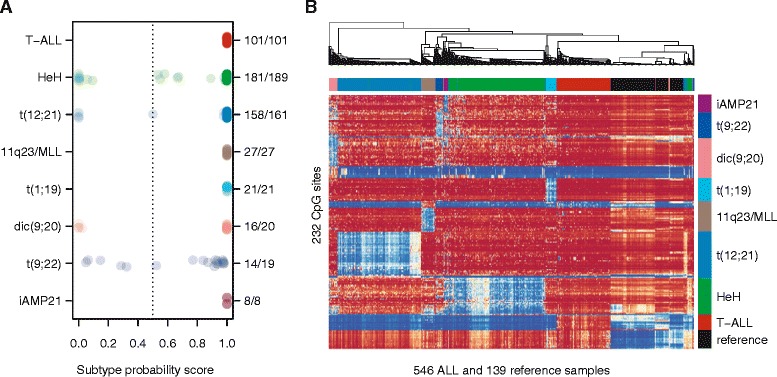
**Prediction of ALL subtypes by consensus CpG sites defined using ALL samples of known subtype. (A)** The estimated subtype probability scores of the 546 patients used to design the classifier. Subtype probability scores are plotted along the horizontal axis. The scores range from 0 to 1, where a score >0.5 is considered a positive classification. The patients are color coded by subtype along the vertical axis. The proportions on the right side of the panel give the number of patients accurately classified by subtype. **(B)** Hierarchical clustering of 546 ALL samples of known subtype and 139 non-leukemic reference samples according to the methylation levels of the 232 autosomal consensus CpG sites. Samples are clustered along the horizontal axis and the consensus CpG sites are clustered along the vertical axis. In the heatmap, blue indicates low, yellow indicates intermediate, and red indicates high methylation levels.

We previously analyzed the genome-wide DNA methylation patterns of 756 primary ALL patients diagnosed between 1996 and 2008 in the Nordic countries [14]. Criteria for selecting patients with established subtypes for the current study included abnormal karyotypes from chromosome banding and/or positive results from targeted FISH or RT-PCR analyses. An overview of the patients included in the study can be found in Additional file 1: Figure S1. In total, 546 patients fulfilled these criteria and were included in the design of the DNA methylation classifier (Table 1, Additional file 2: Table S1). We designed DNA methylation classifiers for the following eight subtypes: T-ALL and the B-cell precursor ALL (BCP-ALL) subtypes HeH, t(12;21), 11q23/*MLL*, t(1;19), dic(9;20), t(9;22), and iAMP21. We also included a classifier for normal blood cells and patient sex to highlight samples with low blast count and to verify the sex of the patients, respectively. We evaluated the performance of the classifier design procedure by cross-validation (Additional file 1: Figures S2–S3). The best performance in terms of sensitivity and specificity was obtained using a set of 246 consensus CpG sites that contained 14-42 CpG sites per ALL subtype (Additional file 1: Figure S4, Additional file 2: Table S2). During cross-validation, on average, 91% of the ALL samples were assigned to one single correct subtype, 3.4% were assigned to multiple subtypes including the correct subtype, and 5.6% were assigned to an incorrect or no subtype (Table 2). When the consensus classifier was trained on the entire data set, it correctly classified 526 out of the 546 samples (95% CI = 515–532 patients). The consensus classifier failed to predict a subtype for as few as 17 patients in the design set, and of these patients, only three were assigned to have an unexpected subtype (Figure 1A, Additional file 2: Table S1).

**Table 1:** **Summary of ALL samples with known subtype used to design DNA methylation-based classifiers**

**Immuno-phenotype**	**Cytogenetic abnormality**	**Fusion gene**	***N***
T-ALL	Various	Various	101
BCP ALL	HeH	-	189
	t(12;21)	*EVT6/RUNX1*	161
	11q23/*MLL*	*MLL*-r	27
	t(1;19)	*TCF3/PBX1*	21
	dic(9;20)	-	20
	t(9;22)	*BCR/ABL1*	19
	iAMP21	-	8

**Table 2:** **Performance of classifiers designed using ALL samples with known subtype**

**Subtype group**	**Mean sensitivity ± SD**	**Mean specificity ± SD**	**Min CpGs (** ***N*** **)** ^**a**^	**Max CpGs (** ***N*** **)** ^**a**^	**Consensus CpGs (** ***N*** **)** ^**b**^
Reference	1.00 ± 0.01	0.99 ± 0.01	13	21	17
T-ALL	0.99 ± 0.02	1.00 ± 0.00	7	17	14
HeH	0.94 ± 0.04	0.95 ± 0.02	23	55	34
t(12;21)	0.97 ± 0.04	0.99 ± 0.01	28	2 263	42
11q23/MLL	0.95 ± 0.11	1.00 ± 0.00	16	27	28
t(1;19)	0.91 ± 0.13	1.00 ± 0.00	9	30	21
dic(9;20)	0.78 ± 0.16	0.99 ± 0.01	24	35	37
t(9;22)	0.70 ± 0.25	0.99 ± 0.01	17	41	23
iAMP21	0.81 ± 0.35	1.00 ± 0.00	11	19	16
Sex	0.98 ± 0.01	1.00 ± 0.01	8	12	14

^a^The minimum and maximum number of CpG sites chosen by each subtype classifier during cross validation.

^b^The number of CpG sites chosen for each subtype in the consensus classifier.

All patients with the iAMP21 subtype (*n* = 8) displayed high prediction scores according to both the iAMP21 and HeH classifiers, while none of the patients with HeH obtained a high score in the iAMP21 classifier. Twenty-six out of 34 (76%) of the consensus CpG sites in the HeH classifier were hypomethylated in the iAMP21 samples at similar levels as in the HeH samples (mean *β*-value iAMP21 = 0.36 and HeH = 0.28, Additional file 2: Table S2). The majority of the consensus sites for HeH were hypermethylated across all the other ALL subtypes (mean *β*-value >0.77) (Figure 1B). It is likely that the consensus classifier for HeH fails to exclude sites where iAMP21 is similar to HeH due to the small number of iAMP21 patients in our study. Furthermore, gains of chromosome 21 are observed in both the iAMP21 and HeH subtypes. Nearly 90% (168/189) of HeH patients have one or more extra copy of chromosome 21, and thus iAMP21 and HeH may share some common biological features due to the increased gene dosage on 21q. Of note, the 10% of atypical HeH patients without extra copies of chromosome 21, as determined by chromosomal banding at diagnosis, were all accurately classified as HeH.

### Blinded validation of ALL subtype classifiers

**Figure 2:**
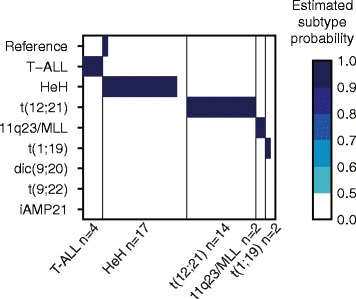
**Subtype prediction of 39 independent validation ALL samples.** Each sample in the validation set is represented as a vertical bar positioned in its corresponding subtype as indicated below the horizontal axis. The color key to the right of the panel shows the estimated subtype probability. A value >0.5 indicates high probability of correct classification. Subtype probability scores <0.5 are not shown.

For independent validation of our DNA methylation classifiers, 39 newly diagnosed ALL patient samples that were not included in the classifier design were analyzed using the 450 k BeadChip (Illumina Inc., San Diego, CA, USA) and subjected to blinded classification. In total, 36 of the 39 (92%) samples were classified correctly (Figure 2). Review of the clinical diagnosis of the three misclassified samples revealed atypical results in the original chromosomal analyses performed at diagnosis (see Additional file 2: Table S3 for detailed information).

### Classification of ALL samples with unknown cytogenetic risk group

**Figure 3:**
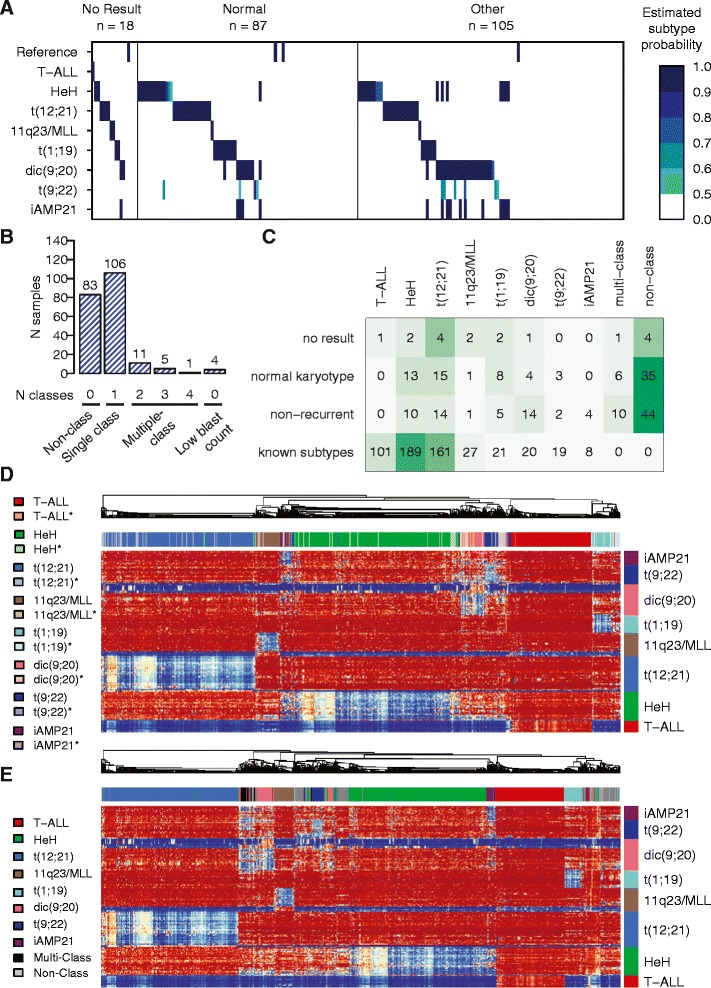
**Classification of ALL samples with undefined cytogenetic subtypes. (A)** Each sample (*n* = 210) is represented as a vertical bar positioned in its corresponding subtype ‘track’ according to its allocation by the classifier. The color key to the right of panel **(A)** shows the estimated subtype probability scores. Probability scores <0.5 are not shown. **(B)** The distribution of probability scores ≥0.5 in the 210 patients. Eighty-three patients were not classified, 106 patients were unequivocally assigned to one subtype, 17 patients were classified into multiple subtype groups, and four patients had high reference scores. **(C)** The distribution of the number of patients with ‘normal’, ‘no result’, and ‘non-recurrent’ karyotypes into subtype-groups. The subtype distribution in the known sample group is also shown. **(D)** Hierarchical clustering of the original 546 ALL patients of known subtype and the patients newly classified as one unequivocal subtype (*n* = 106). Patients are clustered on the horizontal axis and the 215 autosomal subtype-specific consensus CpG sites are clustered on the vertical axis and color-coded by subtype classifier. The darker color indicates samples with previously established cytogenetic subtype, and the corresponding lighter color and asterisk (*) indicates newly classified samples. The color key for the patient samples is shown to the left of the heatmap. In the heatmap, blue indicates low and red indicates high methylation levels. **(E)** Hierarchical clustering and heatmap of the ALL patients of known subtype (*n* = 546), those newly classified and unequivocally assigned to one subtype (*n* = 106), patients without classification (*n* = 83, gray), and patients classified into multiple subtypes (*n* = 17, black). Four patients with suspected low blast count are not shown.

We performed DNA methylation-based subtype classification of 210 BCP-ALL patient samples with no result (*n* = 18), normal (*n* = 87), or non-recurrent karyotype (*n* = 105) (Figure 3A). In total, 106 of the 210 samples were assigned to one of the recurrent subtypes with an estimated class probability of ≥0.50 (Figure 3B). Because all the iAMP21 patients obtained high scores from both the iAMP21 and HeH classifiers in the design set, we counted all patients with this pattern as iAMP21 only. In total, we assigned a subtype to 50 out of 105 patients in the non-recurrent group, 50 out of 87 patients in the group with normal karyotype, and 13 out of the 18 patients in the group with no cytogenetic results available (Additional file 2: Tables S4–S6). The distribution of the newly assigned patients from the normal karyotype group and group of patients with no cytogenetic results was as could be expected in a pediatric ALL population (Figure 3C). The methylation profiles of the newly classified samples closely matched those of the group of original samples used to design the classifier and are referred to as ‘subtype-like’ (Figure 3D, Additional file 1: Figures S5–S12).

A small group of 17 patients classified into two or more groups and are denoted as ‘multi-class’. The most common ‘multi-class’ subtype was double classification as dic(9;20) and t(9;22). Eighty-three patients (~10% of the entire cohort) did not have methylation patterns that are similar to any of the subtype groups, and they were labeled as ‘non-class’. Four patients received high scores in the classifier for non-leukemic reference samples and were excluded from further analysis. According to hierarchical clustering, the patients in the ‘multi-class’ group displayed variable degrees of hypomethylation in the dic(9;20) and t(9;22) consensus CpG sites, which is in agreement with double classification in those subtype groups (Figure 3E, Additional file 1: Figure S13). The ‘non-class’ patients separated into three clusters and did not display strong similarities to any of the other known ALL subtypes.

### Subtype verification

#### Verification by karyotype

All patients in the group with non-recurrent aberrations (*n* = 105) had information from karyotyping performed at diagnosis (Additional file 2: Table S7). We used this information to provide support for the DNA methylation classification (Table 3). Nine out of ten patients with HeH-like methylation profiles had gains of chromosome 21, six of them had 48–49 chromosomes, and three had Down's syndrome (constitutional + chr21). This finding indicates that the methylation status of the genes in the HeH classifier is associated with chromosomal aneuploidy. Thus, these patients may share common biological features although they do not have the >50 chromosomes, which is the criteria used to define HeH as a subtype. All iAMP21-like patients (*n* = 4) and the single patient classified as 11q23-like had aberrations suggestive of, but not conclusive for, the canonical rearrangements in their karyotype data.

**Table 3:** **Summary of subtype verification results**

**Methylation subtype**	**Confirmed subtype** ^**a**^				**Subtype-like** ^**b**^					**Unconfirmed subtype** ^**c**^
	***N***	**Karyotype** ^**d**^	**RT-PCR** ^**e**^	**CNA** ^**f**^	***N***	**Karyotype** ^**d**^	**Negative for fusion gene** ^**g**^	**Novel fusion gene** ^**h**^	**CNA** ^**f**^	***N***
T-ALL*	0/1 (0%)	0	ND	ND	0/1 (0%)	0	NA	ND	ND	1/1 (100%)
HeH*	7/25 (28%)	0	ND	7	18/25 (72%)	8	NA	ND	13	0/25 (0%)
t(12;21)*	4/33 (12%)	1	4	ND	22/33 (67%)	0	19	3	ND	7/33 (21%)
11q23/MLL*	3/4 (75%)	1	ND	3	1/4 (25%)	0	1	ND	ND	0/4 (0%)
t(1;19)*	0/15 (0%)	0	ND	ND	9/15 (60%)	0	9	0	1	6/15 (40%)
dic(9;20)*	3/19 (16%)	0	ND	3	16/19 (84%)	5	NA	0	10	0/19 (0%)
t(9;22)*	0/5 (0%)	0	ND	ND	3/5 (60%)	0	3	ND	ND	2/5 (40%)
iAMP21*	4/4 (100%)	4	ND	4	0/4 (0%)	0	NA	ND	ND	0/4 (0%)

*DNA methylation-based subtype group. Asterisks indicate groups classified by DNA methylation.

^a^Newly classified patients with confirmed verification of their subtype group by at least one verification method.

^b^Newly classified patients negative for the canonical event that defines their subtype and/or non-canonical event.

^c^Newly classified patients without a positive or negative result from one of the verification methods.

^d^Number of patients with a chromosomal aberration observed in karyotyping results determined at diagnosis in NOPHO centers.

^e^Number of patients positive for respective fusion genes by re-analysis by RT-PCR.

^f^The number of patients with chromosomal aberrations discovered by array-based copy number alteration (CNA) that support subtype classification.

^g^The number of subtype-like patients negative for canonical aberrations by targeted analyses (FISH/RT-PCR) at diagnosis.

^h^The number of patients with novel (non-canonical) fusion genes detected.

*Abbreviations*: *NA* not applicable, *ND* not determined.

#### Verification by expressed fusion genes

Targeted analysis using FISH or RT-PCR for *ETV6/RUNX1*, *MLL* rearrangements, *PBX1/TCF3*, and *BCR/ABL1* had been performed for only 57% of the subtype-like patients at the time of diagnosis. Therefore, it is likely that many of the newly classified patients actually harbor the canonical translocations that define the group they were assigned to by our DNA methylation classifier. Re-analysis by RT-PCR for the *ETV6/RUNX1* fusion transcript in RNA taken at diagnosis from eight randomly selected t(12;21)-like patients with available RNA showed that half of them were positive for *ETV6/RUNX1* (Additional file 2: Table S8).

We performed RNA-seq of 17 patients with available high quality RNA for whom cytogenetic subtype information from ALL diagnosis and the results obtained by the DNA methylation classifier did not agree. In nine out of these 17 patients, we detected expressed fusion genes (Table 4, Additional file 2: Table S9). Three previously unknown fusion genes t(20;21)*RUNX1/ASXL1*, t(7;12)*ETV6/CBX3*, and t(3;12)*ETV6/AK125726* were identified in patients with t(12;21)-like methylation profiles. We found that several of the patients assigned to the ‘multi-class’ group according to the DNA methylation classifier harbored fusion genes with *PAX5* as one of the fusion partners, including the known t(9;12)*PAX5/ETV6* and inv(9p13.2)*PAX5/ZCCHC7* fusion genes previously reported in ALL [19-21]. We also identified a new fusion gene, t(9;14)*PAX5/ESRRB*, which to our knowledge has not been previously reported. In an infant patient with HeH in the validation cohort who was misclassified as ‘non-class’ (ALL_validation_20 in Table 4), we identified an additional novel fusion gene, t(5;15)*BRD9/NUTM1.*

**Table 4:** **Fusion gene screening by RNA sequencing**

**Sample ID**	**Subtype**	**Methylation subtype** ^**a**^	**FISH/PCR**	**Read pairs**	**Fusion gene**
ALL_176	Non-recurrent	t(1;19)*	t(1;19) Not tested	84.3 M	No fusion detected
ALL_392	Normal	t(1;19)*	t(1;19) Negative	94.4 M	No fusion detected
ALL_390	Normal	t(1;19)*	t(1;19) Negative	102.6 M	No fusion detected
ALL_11	No result	t(12;21)*	t(12;21) Not tested	99.5 M	t(20;21)*RUNX1/ASXL1*
ALL_106	HeH	t(12;21)*	t(12;12) Negative	57.3 M	t(7;12)*CBX3/ETV6*
ALL_495	Normal	t(12;21)*	t(12;21) Negative	32.1 M	t(3;12)*ETV6/AK125726*
ALL_391	Non-recurrent	dic(9;20)*	NA	98.6 M	No fusion detected
ALL_58	Non-recurrent	Non-class	NA	89.0 M	No fusion detected
ALL_61	Normal	Non-class	NA	101.8 M	No fusion detected
ALL_Validation_20	HeH	Non-class	NA	19.0 M	t(5;15)*BRD9/NUTM1*
ALL_313	Non-recurrent	Multi-class	NA	116.7 M	No fusion detected
ALL_619	Non-recurrent	Multi-class	NA	84.3 M	t(9;14)*PAX5/ESRRB*
ALL_403	Non-recurrent	Multi-class	NA	95.2 M	inv(9)*PAX5/ZCCHC7*
ALL_246	Non-recurrent	Multi-class	NA	120.5 M	t(9;12)*PAX5/ETV6*
ALL_485	Non-recurrent	Multi-class	NA	90.6 M	t(9;12)*PAX5/ETV6*
ALL_373	Non-recurrent	Multi-class	NA	96.2 M	del(X)*P2RY8/CRLF2*
ALL_497	Non-recurrent	Multi-class	NA	89.7 M	No fusion detected

*Patients with DNA methylation patterns similar to those of the recurrent ALL subtypes.

^a^Subtype determined by DNA methylation-based classification.

*Abbreviation*: *NA* not applicable.

#### Verification by copy number analysis

Since the 450 k BeadChip assay uses the same reaction principle as SNP genotyping arrays, the 450 k data can be used to detect copy number alterations (CNAs) [22-24]. CNA analysis was applied to identify large-scale chromosomal gains and losses to support the subtype classification by DNA methylation in the subtype-like patients, in whom unbalanced large scale chromosomal alterations are expected to occur, such as in HeH, t(1;19), dic(9;20), and iAMP21 (Table 3, Additional file 2: Table S7).

We observed >50 chromosomes in six out of 15 of the HeH-like patients with ‘normal’ or ‘no result’ in the karyotype analysis, suggesting that these patients did in fact harbor aneuploidies that were undetected at diagnosis (Additional file 1: Figure S14). We also found evidence for amplification of chromosome 21q, which is consistent with the iAMP21 subtype in each of the four iAMP21-like patients (Additional file 1: Figure S15). In three out of the 21 dic(9;20)-like patients and in one multi-class patient, we found deletions of chromosome 9p and 20q, which confirm that these four patients harbor dic(9;20). Thirteen out of the 20 remaining dic(9;20)-like patients and 11 out of the 17 ‘multi-class’ patients displayed deletions of various sizes on 9p but lacked 20q deletions (Additional file 1: Figure S16).

We found a breakpoint in the *TCF3* locus in one of the t(1;19)-like patients (Additional file 1: Figure S17). The remaining 18 t(1;19)-like patients showed no evidence of CNAs on chromosomes 1 or 19, which does not exclude that these patients harbor balanced translocations, which are common in this subtype. Although t(9;22) results in a balanced re-arrangement that cannot be detected by CNAs, we screened the t(9;22)-like patients for *IKZF1* deletions which are known to occur in patients with the *BCR/ABL1* fusion gene and *BCR/ABL1*-like gene expression patterns [25]. In three out of the five t(9;22)-like patients, we detected intragenic *IKZF1* deletions or iso(7q), resulting in hemizygous loss of *IKZF1* (Additional file 1: Figure S18).

### Clinical outcome of the newly classified ALL patients

The clinical features of the newly classified patients, including age, white blood cell count at diagnosis, central nervous system involvement, and outcome were similar to those of the original patients with known subtypes (Table 5). No significant differences in the cumulative incidence of relapse were detected between the newly classified and previously established patient groups (Additional file 1: Figures S19–S20). The multi-class group had an overall favorable prognosis (one relapse in 17 patients), despite the fact that the patients in this group had a median age of diagnosis of 10 years.

**Table 5:** **Clinical characteristics of ALL patients of known and newly classified subtype**

**Subtype**	***N*** **(%)** ^**a**^	**Sex M:F**	**Age median (range)**	**WBC median (range)**	**CNS,** ^**b**^ **n/y/u**	**CIR** ^**c**^	**NOPHO protocol** ^**d**^				**EsPhALL** ^**e**^	**Infant** ^**f**^	**Other/NA** ^**g**^
							**SR**	**IR**	**HR**	**Ph+**			
T-ALL	101 (0.18)	2.9	9 (1–17)	138 (1–788)	89/12/0	0.20	0	2	86	1	0	0	12
T-ALL*	1 (0.00)	NA	11 (NA)	2 (NA)	1/0/0	0.00	0	0	1	0	0	0	0
HeH	189 (0.35)	1.2	3 (1–17)	9 (0–131)	187/2/0	0.16	82	79	22	0	0	0	6
HeH*	25 (0.12)	2.1	4 (1–14)	9 (1–91)	24/1/0	0.28	8	10	3	0	0	0	4
t(12;21)	161 (0.29)	1.1	4 (1–15)	14 (1–226)	159/0/2	0.22	68	65	24	0	0	0	4
t(12;21)*	33 (0.16)	1.1	4 (0–15)	11 (1–136)	33/0/0	0.24	12	11	6	0	0	1	3
11q23/MLL	27 (0.05)	0.7	0 (0–15)	193 (2–986)	25/1/0	0.41	0	1	11	0	0	14	1
11q23/MLL*	4 (0.02)	4.0	1 (0–1)	81 (38–1,255)	4/0/0	0.50	0	1	2	0	0	1	0
t(1;19)	21 (0.04)	0.8	11 (1–15)	35 (6–159)	20/1/0	0.14	0	3	17	0	0	0	1
t(1;19)*	15 (0.07)	0.9	7 (1–15)	10 (5–222)	14/1/0	0.07	1	9	3	0	0	0	2
dic(9;20)	20 (0.04)	0.4	2 (1–15)	50 (4–336)	19/1/0	0.25	4	5	10	0	0	0	1
dic(9;20)*	19 (0.09)	1.7	4 (1–18)	12 (1–164)	19/0/0	0.21	3	11	2	0	0	0	3
t(9;22)	19 (0.03)	2.2	10 (2–14)	61 (3–246)	17/2/0	0.47	0	0	1	6	12	0	0
t(9;22)*	5 (0.02)	4.0	12 (4–15)	102 (13–146)	5/0/0	0.40	0	0	1	0	0	0	4
iAMP21	8 (0.01)	7.0	9 (5–17)	5 (2–62)	8/0/0	0.38	2	3	1	0	0	0	2
iAMP21*	4 (0.02)	0.3	9 (7–16)	9 (4–23)	4/0/0	0.50	1	2	0	0	0	0	1
Multi-class	17 (0.03)	0.9	10 (3–17)	23 (1–106)	16/1/0	0.06	1	6	2	0	0	0	8
Non-class	83 (0.15)	1.5	9 (0–18)	20 (1–274)	80/3/0	0.23	13	30	25	0	0	3	11

*Patients with subtype determined by DNA methylation-based classification.

^a^Proportion of the total number of patients in the known subtype groups (*n* = 546) and newly classified patients (*n* = 210).

^b^Central nervous system involvement.

^c^Cumulative incidence of relapse (relapse as event of interest and other events as censoring).

^d^Number of patients treated on NOPHO-ALL treatment protocols.

^e^Number of patients treated according to the EsPhALL protocol.

^f^Number of patients treated according to the Interfant-99 or Interfant-06 protocols.

^g^Number of patients treated on other protocol or information not available.

*Abbreviations*: *WBC* white blood cell count at diagnosis, *n/y/u* no/yes/unknown, *SR* standard risk, *IR* intermediate risk, *HR* high risk, *Ph +* Philadelphia positive.

### Down's syndrome ALL

Nineteen BCP-ALL patients with Down's syndrome (DS-ALL) were included in our study. These patients were not classified separately from ALL patients without DS. Two DS-ALL patients had t(12;21) and one had t(9;22). Each of these three patients was classified correctly according to their cytogenetic subtype. Eight DS-ALL patients had the karyotype ‘other’, seven had a ‘normal’ karyotype, and one had ‘no result’. Only four of the DS-ALL patients were classified as HeH-like, and three were confirmed to have 48–49 chromosomes at diagnosis by chromosomal banding or array-based CNA detection. The chromosomal gains included +14, +17, and + X, which are the typical chromosomal gains observed in HeH in addition to +21c. Additional details about the classification of DS-ALL patients can be found in Additional file 2: Table S7.

### Annotation of the consensus CpG sites in the ALL subtype classifier

Remarkably, none of the consensus CpG sites in the classifier were located in the genomic regions harboring the subtype-specific cytogenetic aberrations. For example, none of the consensus CpG sites for the iAMP21 subtype were located on chromosome 21, none of the CpG sites for the t(12;21) subtype were located on chromosomes 12 or 21, and none of the CpG sites defining the 11q23/MLL, dic(9;20), and t(1;19) subtypes were on chromosomes 11, 9, 20, 1, or 19, respectively.

Over 90% of the consensus CpG sites for each the BCP ALL subtypes were hypomethylated (median *β*-value 0.19) in the patients belonging to the respective subtypes, while all other patients were highly methylated. The CpG sites in the T-ALL classifier were hypermethylated in T-ALL patients (median *β*-value 0.92) and hypomethylated in the BCP ALL patients (median *β*-value 0.04). Over 95% of the consensus CpG sites were annotated to protein coding genes, and the majority (87%) of them were located outside CpG islands (Additional file 2: Table S2). Several of the genes highlighted in our classification procedure are associated with a somatic mutation or differential DNA methylation or gene expression patterns in ALL subtypes such as *DDIT4L*(4q23) in HeH [14,26], *CBFA2T3*(16q24), *TCFL5*(20q13.33), *DSC3*(18q12.1), and *EPOR*(19p13.3-p13.2) in t(12;21) [12,14,25-29], *MBNL1*(3q25) and *ZEB2* (2q22.3) in 11q23/MLL [25,30], and *NT5C2*(10q24.32) and *PON2*(7q21.3) in t(9;22) [26,27,31,32]. However, most of the genes that we identified with CpG methylation that was characteristic of specific ALL subtypes have no previously known function or connection with ALL.

## Discussion

We present a method for the identification of recurrent cytogenetic abnormalities in patients with ALL using DNA methylation profiling. Our DNA methylation classifier that consists of only 246 CpG sites is able to accurately detect the subtype of primary ALL samples. Nearly 50% of 210 ALL patients in our study that had not previously been assigned to a recurrent subtype group at diagnosis displayed DNA methylation patterns that are similar to those of the eight recurrent subtypes of ALL investigated in the present study. Thus, DNA methylation analysis could complement current cytogentic and molecular biological analyses applied in routine diagnosis of ALL to allow stratification of a larger number of ALL patients into risk-based treatment groups. Verification of our DNA methylation-based analyses suggests that at least 21 out of the 106 patients classified by DNA methylation harbor one of the canonical aberrations that define an ALL subtype, which were not detected at the time of diagnosis. Since our cohort of ALL patients included samples diagnosed as early as 1996, the fact that the canonical aberrations were not detected at diagnosis could be due to technical limitations of the methods applied at that time.

In contrast to traditional methods used for diagnostics, which require >1 ug of RNA or intact dividing cells, analysis of DNA methylation using the 450 k BeadChip requires only 250 ng of DNA, which is useful for cases where little material is available, especially for biobanked samples. A more targeted diagnostic test than the 450 k BeadChip could be constructed for routine use to analyze the limited number of CpG sites that constitutes the classifier. On the other hand, the use of the 450 k BeadChip could be an advantage as the CpG site content of the classifier might be altered when novel ALL subtypes have been defined.

In our study, we found several new fusion genes in subtype-like patients that involve the same chromosomes and genes as the known fusion genes that define the known subtypes of ALL, which appear to result in the similar DNA methylation patterns. The patients harboring non-canonical gene fusions include *ETV6*, *RUNX1*, or *PAX5*. This observation indicates that alterations that affect either one or both of the gene fusion partners may influence the DNA methylation patterns of the CpG sites in our classifier. The newly identified fusion genes in patients with t(12;21)-like methylation profiles include *CBX3/ETV6,* and *RUNX1*/*ASXL1*, and notably both *CBX3* and *ASXL1* are known to be mutated in ALL and in AML, respectively [31,33]. Furthermore, the patients that we classified into multiple subtype groups display the previously unreported *PAX5/ESRRB* fusion gene and the *PAX5/ZCCHC7* and *PAX5/ETV6* fusions that occur in approximately 1% of ALLs [19-21]. The high prevalence of *PAX5* fusions in this group raises the question of whether these patients comprise a biologically and clinically distinct subgroup. We also discovered an unexpected novel fusion gene involving *BRD9/NUTM1* in an infant patient with HeH who did not classify into any subtype group. BRD9 is required for the oncogenic properties of the *MLL*-fusion proteins [34], which are common in infant ALL patients. A similar fusion gene, *BRD4/NUTM1*, defines a lethal subtype of midline carcinoma [35,36]. These observations warrant further investigation in additional pediatric ALL patients to determine their prognostic or potential therapeutic value.

The translocations resulting in the expression of a fusion protein may modulate DNA methylation patterns via aberrant repression or activation of downstream genes. This hypothesis is supported by the observation that the majority (85%) of CpG sites in the consensus classifier are hypomethylated, by previous reports of aberrant gene expression in the subtype for which they were selected [14,18,25-28], and because the subtype-specific sites are spread out across all autosomes and are not clustered near the physical translocation breakpoints. One patient (Validation_ALL_39) was positive for t(1;19) with FISH and negative for expression of *TCF3/PBX1* due to deletion of the *TCF3* gene in the translocation. Consequently, this patient failed to classify as a t(1;19). The alteration of gene expression cascades due to the activity of the fusion proteins may be essential for the DNA hypomethylation pattern in patients with t(1;19) and the other translocations, which presumably occur in the early stages of leukemic transformation and are maintained through cellular division and clonal evolution.

The reason behind the striking similarities in DNA methylation in patients with HeH remains a mystery. Because the DNA methylation profiles are so similar in patients harboring various combinations of chromosomal gains, the methylation changes may predate the gains in chromosome number. Yeoh and colleagues reported that most of the genes whose expression patterns define HeH are on chromosomes 21 or X [28]. Although we did not analyze the X chromosome due to its inactivation in females, the consensus CpG sites for HeH were distributed across the different autosomes and only two of the CpG sites were on chromosome 21. One of them was in the *CLDN14* gene in the Down's syndrome breakpoint region, and the second CpG site was located in a non-coding RNA gene on 21q22.3. Our results indicate that the HeH-like patients with 47–50 chromosomes are a biological group with similar etiology and clinical outcome as those with >50 chromosomes. As more ALL genomes are sequenced, it will be interesting to see if there are recurrent somatic mutations and/or cryptic genomic rearrangements that provide a unifying cause underlying the HeH subtype.

We recognize that several more recently identified subtypes characterized by additional aberrations of *IKZF1*(7p12.2), *ERG*(21q22.3), *CRLF2*(Xp22.3 and Yp11.3), and translocations involving tyrosine kinase genes were not included in the present study [1,2,37]. The prevalence of such aberrations is under investigation in Nordic ALL patients [38,39]. When these data become available, it will be possible to determine if the patients harboring these aberrations form distinct subgroups based on their DNA methylation profiles.

## Conclusions

Together with response to induction therapy, genetic aberrations are among the most important prognostic factors in pediatric ALL. Our findings indicate that DNA methylation profiling can contribute to reduction of the heterogeneity in undefined ALL patient groups and can potentially be implemented for diagnostics of ALL and possibly other types of hematological cancers. Follow-up studies of findings where DNA methylation and cytogenetic aberrations do not agree provide an interesting approach for the discovery of previously unrealized chromosomal aberrations in ALL. Annotation of the CpG sites that constitute the subtype classifier highlights genes that are known to be relevant for ALL, which suggests a functional role for methylation of these sites but also genes with no known function in ALL are highlighted for further studies.

## Methods

### Clinical diagnostic analysis of ALL samples

Bone marrow aspirates or peripheral blood samples were collected at diagnosis from 756 population-based pediatric ALL patients enrolled between 1996 and 2010 on the Nordic Society of Pediatric Hematology and Oncology (NOPHO), EsPhALL, or Infant treatment protocols (Additional file 2: Table S10) [4,40,41]. Diagnoses were established by analysis of leukemic cells with respect to morphology, immunophenotype, and cytogenetics. HeH was defined as 51–67 chromosomes per cell [42]. FISH or RT-PCR analyses were used to screen for the following translocations: t(12;21)(p13;q22)[*ETV6/RUNX1*], t(9;22)(q34;q11)[*BCR/ABL1*], and t(1;19)(q23;p13.3)[*TCF3/PBX1*]. FISH or Southern blot analyses were used to identify *MLL* rearrangements, more than three copies of *RUNX1* by FISH define iAMP21, and high resolution SNP arrays and/or FISH were used to detect dic(9;20) aberrations [43,44]. The study was approved by the Regional Ethical Review Board in Uppsala, Sweden and was conducted according to the guidelines of the Declaration of Helsinki. The patients or their guardians provided informed consent.

### DNA methylation assay and samples

Genome-wide DNA methylation data for ~450,000 CpG sites was generated as previously described [14]. A total of 546 out of 756 patients were determined to have established subtype by chromosome banding and/or positive results from targeted FISH and RT-PCR analyses and were included in the design of the DNA methylation classifier (Table 1, Additional file 2: Table S1). Thirty-nine blinded DNA samples were obtained from newly diagnosed ALL cases as an independent validation set (Additional file 2: Table S3). The reference panel for determining samples with low leukemic blast content consisted of remission bone marrow aspirates from pediatric ALL patients (*n* = 86) and fractionated blood cells from healthy donors (*n* = 51) [14].

In total, 210 patients included in the current study did not belong to one of the canonical subtypes according to results from chromosomal banding or targeted assays performed at ALL diagnosis. Patients denoted as ‘non-recurrent’ harbored non-recurrent aberrations (*n* = 105, Additional file 2: Table S4). Patients designated as ‘normal’ (*n* = 87) displayed normal karyotypes and were negative in the targeted assays (Additional file 2: Table S5). Patients designated as ‘no result’ failed in the cytogenetic analysis (*n* = 18, Additional file 2: Table S6).

### Predictive modeling of ALL subtypes using DNA methylation

Methylation-based classifiers were designed to distinguish between ten pairs of groups: ALL against reference samples, female against male, and each of the eight subtypes T-ALL, HeH, t(12;21), 11q23/*MLL*, t(1;19), dic(9;20), t(9;22), and iAMP21 against a background of the other ALL subtypes. The sex classifier was trained on all chromosomes except Y, and the other classifiers were trained on autosomes only. The male-versus-female classifier was implemented to highlight sample mix-ups. The classifiers were created using Nearest Shrunken Centroid (NSC) classification [45]. The NSC modeling procedure consisted of a feature selection step and a training step (Additional file 1: Figure S2). In the feature selection step, fivefold cross-validation was repeated five times. CpG sites selected during the NSC training process in 17/25 cross validation folds were selected as ‘consensus CpG sites’ (Additional file 1: Figure S3). The performance of the consensus classifier was evaluated using external cross-validation (Additional file 1: Figure S2). Additional details on the classification procedure can be found in Additional file 1.

### Analysis of copy number alterations

CNA data was generated from raw signal intensities extracted from Genome Studio (Illumina Inc., San Diego, CA, USA). For each probe, the intensities were summed (methylated + unmethylated signals) and subjected to quantile normalization using the preprocessCore package in R [46]. Log2 ratios were calculated by dividing the normalized intensity by the mean intensity across the non-leukemic reference cells. CNAs were detected by plotting the log2 ratios in the integrative genomics viewer (IGV) [47].

### Analysis of fusion genes

One microgram of total RNA was converted to cDNA using the Superscript III kit (Life Technologies, Carlsbad, CA, USA) and subjected to RT-PCR for the fusion transcript *ETV6/RUNX1* using the probe set ENF301-ENPr341-ENR361 (Life Technologies, Carlsbad, CA, USA). Strand-specific RNA-sequencing libraries were generated from 1 μg total RNA with the ScriptSeq v1.2 kits (Epicentre, Madison, WI, USA), followed by sequencing on a HiSeq2000/2500 or MiSeq instrument (Illumina Inc., San Diego, CA, USA). Gene fusions were detected using the FusionCatcher software [48]. Details about the fusion gene analysis can be found in Additional file 1.

### Availability of supporting data

Methylation data are available at the Gene Expression Omnibus under series GSE49031. The R-code is available at Github (https://github.com/Molmed/Nordlund-ALL-subtyping).

## Additional files

Additional file 1:
**Supplementary methods and supplementary Figures S1–S20.**
Additional file 2:
**Supplementary Tables S1–10.**

## Abbreviations

5mC: 5-Methyl cytosine
ALL: Acute lymphoblastic leukemia
BCP ALL: B-cell precursor ALL
CNA: Copy number alteration
FISH: Fluorescent *in situ* hybridization
HeH: High hyperdiploidy
iAMP21: Intrachromosomal amplification of chromosome 21
PCR: Polymerase chain reaction
T-ALL: T-cell ALL
