# Supplementary material for: DNA methylation-based subtype prediction for pediatric acute lymphoblastic leukemia
Source: Clin Epigenetics. 2015 Feb 17;7(1):11. doi: 10.1186/s13148-014-0039-z (PMC4343276; doi:10.1186/s13148-014-0039-z)

Supplementary Methods

Predictive modeling of ALL subtype using DNA methylation

Methylation based classifiers were designed to identify 10 different pairs of classes: ALL vs reference samples, each of the eight subtypes (T-ALL, high hyperdiploidy (HeH), t(12;21, 11q23/MLL, t(1;19), dic(9;20), t(9;22), iAMP21 against a background of the other subtypes), and sex. The sex classifiers were trained on CpG sites across all chromosomes except Y and the other classifiers were trained on autosomal CpG sites only.

The design procedure consisted of two steps: a feature selection step that reduced the entire array to a small set of sites and a training step that produced the final classifiers used for prediction, called the consensus classifier (**Supplementary Figure S2**). In the feature selection step, the dataset was split into multiple pairs of design and test sets using repeated cross validation (CV, 5 replicates of 5 folds each), and one nearest shrunken centroid classifier (NSC)[1] was fitted for each pair of classes within each fold. In addition to the standard NSC fitting algorithm, a preprocessing step was included that discarded and CpG sites with more than 10% missing values and CpG sites with a difference in class-wise mean β-value <20%. This was included to remove CpG sites with many missing values and to remove CpG sites with small differences that are unlikely to be of biological relevance.

Preprocessing and NSC fitting resulted in classifiers that included a small number of CpG sites as basis for classification. CpG sites that were selected in at least 17 of the 25 CV folds (τ), referred to as the consensus sites were used to fit one final NSC classifier per pair of classes (**Supplementary Figure S3**). Furthermore, external CV was used to evaluate whether to let all final classifiers use all consensus CpG sites or to limit the final classifiers to only use the consensus sites of their own classes. Allowing the final classifiers to use all consensus CpG sites resulted in fewer incorrect class calls in the smaller classes, and thus this method was chosen for the final classification procedure (**Supplementary Figure S2**).

To estimate the uncertainty of the estimated error rates, 95% credible intervals (also known as Bayesian confidence intervals) were calculated. These were computed numerically as the 2.5% and 97.5% quantiles of the error rate's posterior probability density function *p(q|kt,Nt)*, which in turn was calculated using Bayes theorem:

$$p(q|k_t, N_t) = \frac{P(k_t|q, N_t)p(q)}{P(k_t|N_t)} = \frac{q^{k_t}(1-q)^{N_t-k_t}p(q)}{\int_0^1 q^{k_t}(1-q)^{N_t-k_t}p(q)dq}$$

where q is a the error rate of a classifier that produced kt errors on a test set of size Nt, and $p(q)=1$.

RNA-sequencing and fusion gene detection

RNA was quantified using a Qubit 2.0 Fluorometer (Invitrogen) and RNA quality was measured on a Bioanalyzer 2100 using the RNA 6000 Nano Assay (Agilent). All RNA samples had RIN values > 7. Prior to library preparation, ribosomal RNA was depleted from 1 µg of total RNA using the Ribo-Zero rRNA Removal Kit (Epicentre). Strand-specific libraries for RNA-sequencing (RNA-seq) were prepared using the ScriptSeq v2 RNA-Seq Library Preparation Kit (Epicentre). Libraries were sequenced with paired-end 50 bp reads on the HiSeq2000 or paired-end 75 pb on a MiSeq instrument (Illumina) to a minimum read depth of 20 million read-pairs per library.

The FusionCatcher software version 0.99.1 beta (http://code.google.com/p/fusioncatcher/) was used to detect novel/known fusion genes, translocations, and chimeras in the RNA-seq data.[2] The following parameters were used: --assembly --explore-2.

Candidate fusions with very high probabilities of being false positives according to FusionCatcher documentation and denoted in the "Fusion_description" heading (banned, conjoining, ctd_gene, distance1000pb, distance 10kbp, fully_overlapping, healthy, mt, pair_pseudo_genes, rp11_gene, rp_gene, rrna, same_strand_overlapping, short_distance, similar_reads, similar_symbols) were removed from analysis. Only in frame fusions were retained. Any fusions involving one of the known ALL fusion genes (PAX5, ETV6, RUNX1, PBX1, TCF3, BCR, ABL1, or MLL) were retained for further analysis even if it was excluded according to the parameters mentioned above. All remaining fusions were verified by aligning all raw reads to the genome (human_g1k_v37) using Tophat2 (2.0.4)[3] as implemented in the "Piper" pipeline for RNA-seq data analysis (https://github.com/Molmed/piper). The following parameters were used for Tophat2: --library-type fr-secondstrand --GTF [Ensembl GRCh37 release 66] -p 8 --keep-fasta-order. Fusions were manually verified by viewing the aligned reads supporting the fusion using IGV.[4] All fusions without at least 4 uniquely aligned read pairs spanning the breakpoint were excluded.

Verification of expressed fusion genes

To verify the presence of expressed fusion genes, detailed analysis of cytogenetic data and/or verification by PCR and Sanger sequencing was performed for selected samples. First, when available, cytogenetic data was used to search for chromosomal aberrations supporting observed fusion events in the RNA-sequencing data (**Supplementary Table S7**). Second, PCR primers were designed using Primer3 software (http://primer3.ut.ee/) to amplify from the exon with supporting fusion reads or the next expressed upstream exon of the 3' fusion gene and the exon with the read pairs supporting the fusion or next expressed downstream exon of the 5' fusion gene. All primer sequences can be found in **Supplementary Table S8.** 250 ng of RNA was reverse transcribed into in cDNA using the Superscript III First-Strand Synthesis SuperMix for qRT-PCR (Invitrogen). 12.5 ng of cDNA was amplified in the PCR

reaction using 0.2mM primers and Taq polymerase (Invitrogen) in a 25 ul reaction. The PCR products were sequenced using BigDye Terminator v3.1 chemistry and an Applied Biosystems 3730XL DNA sequencer. Resulting sequences were mapped back to the genome using BLAT (http://genome-euro.ucsc.edu/index.html).

References

1. Tibshirani R, Hastie T, Narasimhan B, Chu G. Diagnosis of multiple cancer types by shrunken centroids of gene expression. *Proc Natl Acad Sci U S A* 2002 May 14; **99**(10)**:** 6567-6572.

2. Edgren H, Murumagi A, Kangaspeska S, Nicorici D, Hongisto V, Kleivi K*, et al.* Identification of fusion genes in breast cancer by paired-end RNA-sequencing. *Genome biology* 2011; **12**(1)**:** R6.

3. Kim D, Pertea G, Trapnell C, Pimentel H, Kelley R, Salzberg SL. TopHat2: accurate alignment of transcriptomes in the presence of insertions, deletions and gene fusions. *Genome biology* 2013 Apr 25; **14**(4)**:** R36.

4. Thorvaldsdottir H, Robinson JT, Mesirov JP. Integrative Genomics Viewer (IGV): high-performance genomics data visualization and exploration. *Briefings in bioinformatics* 2013 Mar; **14**(2)**:** 178-192.

Supplementary Figures

Supplementary Figure S1:

Flow chart of the classification design and validation.

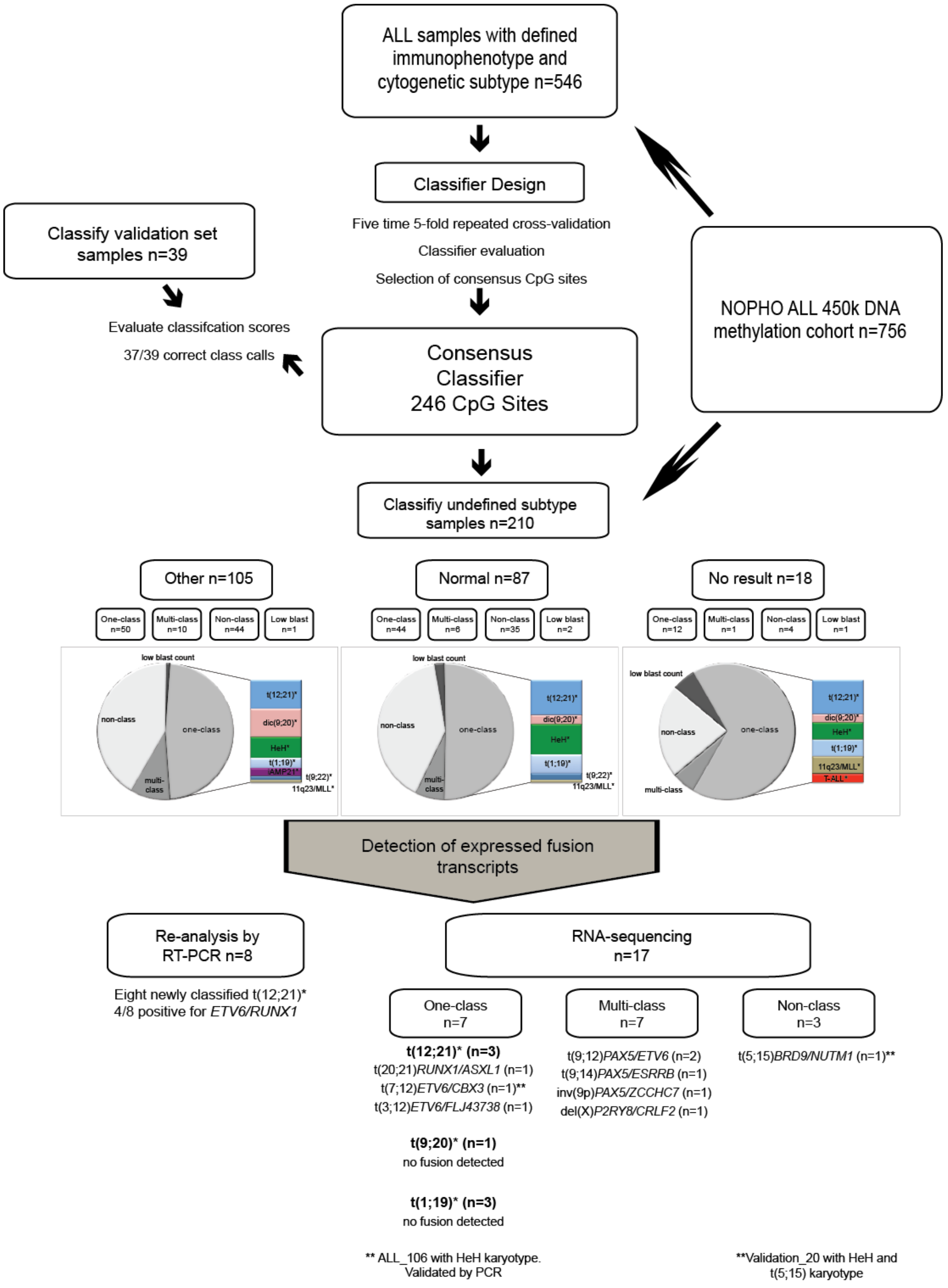

Supplementary Figure S2: *The design procedure for constructing the consensus classifiers*.

a) Classifier training procedure. Rather than modeling all subtypes simultaneously as a multi-class problem, they were modeled as a series of binary "one vs the rest" problems. In order to find the most coherent CpG-sites among many with highly similar content, a series of nearest shrunken centroid (NSC) classifiers were trained on 25 randomly selected subsets of the training data (performed separately for each subtype). Sites choosen at least τ times were defined as the subtype-specific consensus sites. All training data was then used to create subtype-specific classifiers using the consensus sites only. See Supplementary Figure S3 for details on the parameter selection. b) Performance estimation procedure. The entire dataset was randomly divided into 25 pairs of training and test sets (5 replicates of balanced 5-fold cross-validation). One set of subtype-specific classifiers was trained on each training set and its performance was evaluated using its corresponding test set. c) Final classifier training. The entire dataset was used to train the final classifiers that were used to predict the subtypes of the samples with unknown subtype and the blinded validation samples.

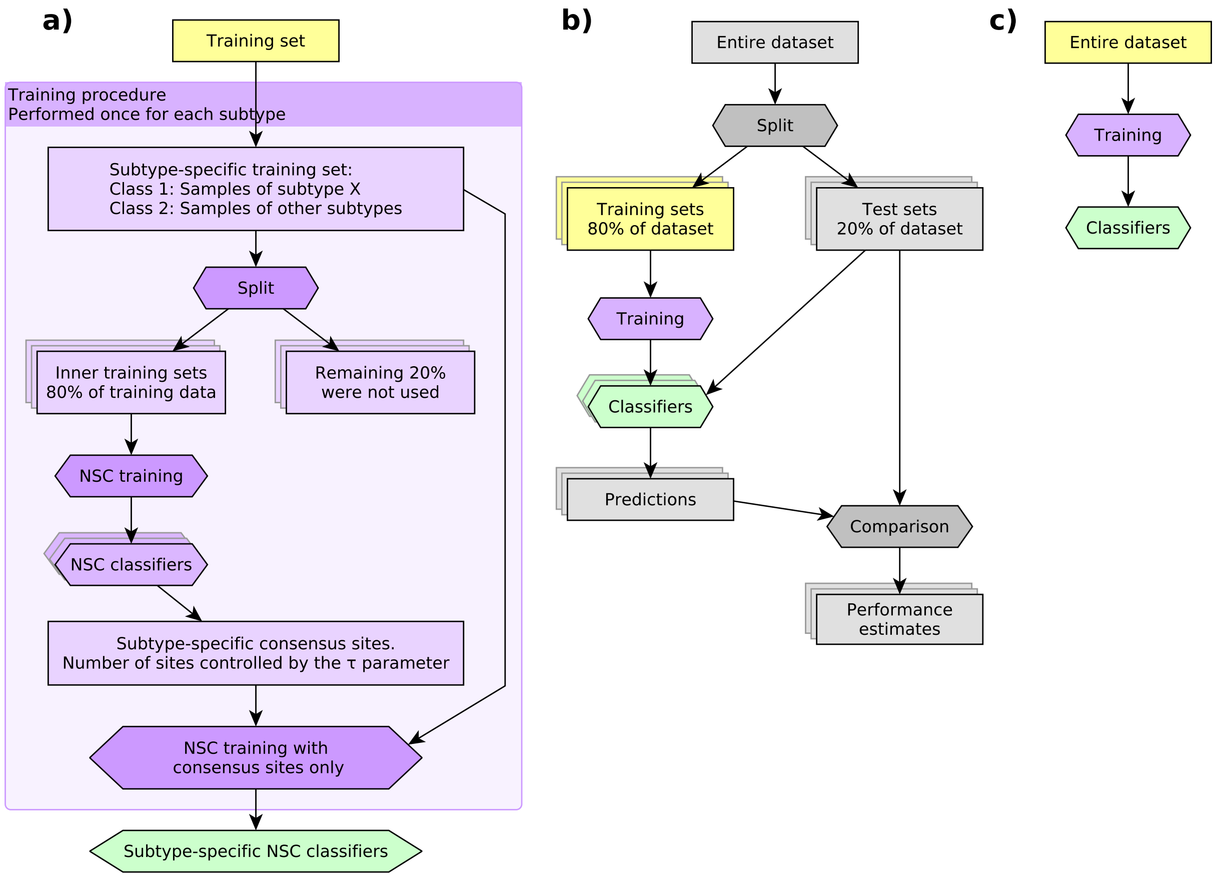

Supplementary Figure S3: *Parameter tuning results.*

When training classifiers as described in Supplementary Figure S2A, there are two parameter choices to be made: 1) The value of τ which controls the size of the consensus set and 2) whether to let the subtype-specific classifers use the consensus sites for all subtypes combined (red) or only the ones selected for its own subtype (blue). All combinations of parameter choices were evaluated using the method outlined Supplementary Figure S2B. a) Overall performance was measured by error rate, where an error was defined as classifying an ALL sample as reference, a reference sample as ALL, or to incorrectly class the subtype of an ALL sample. Since few of the known ALL samples belong to multiple subtypes, a double assignment was also considered an error. b) Plots of the mean sensitivity over the 25 cross validation folds. c) Plots of the mean specificity over the 25 cross validation folds.

Taken together (a-c), the performance was relatively insensitive to the choice of parameter values. For the subtypes with fewer biological replicates, we observed a slight increase in specificity with the combined method, which resulted in fewer false positives. Thus the combined method used for the final classifier. A final value of τ=17 was manually selected as a trade-off between consensus set redundancy and overall and class-wise performance.

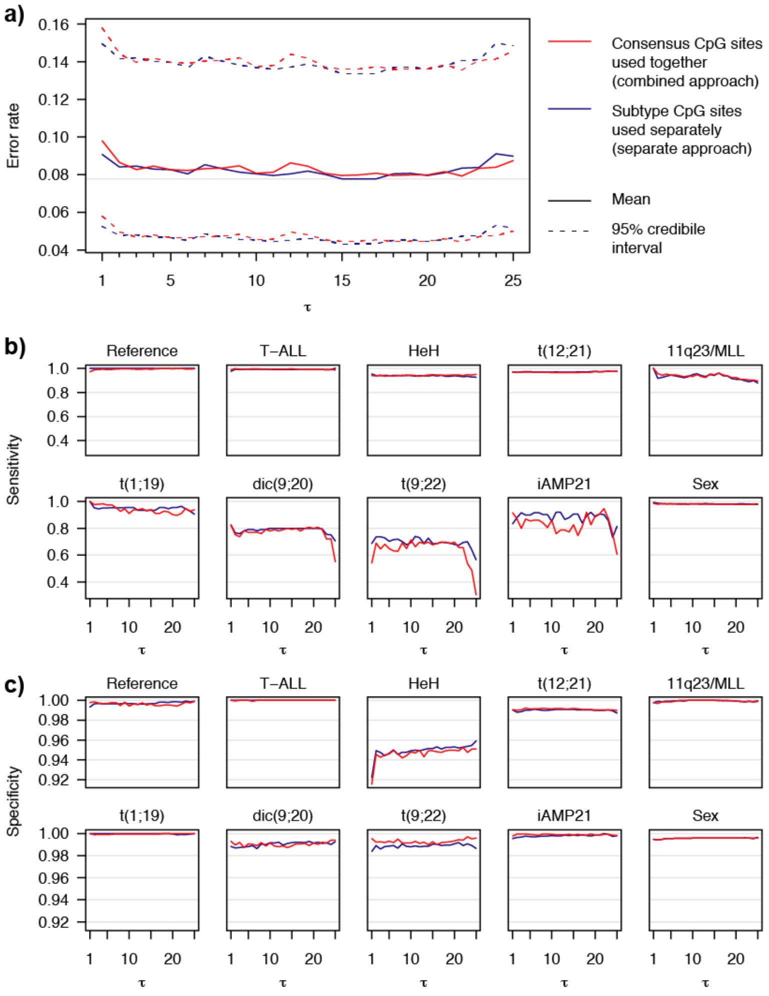

Supplementary Figure S4: *ROC curves.*

The curves show the average performance across the cross-validation replicates using τ=17 (solid) and the 95th percentile (dashed) to illustrate the variability of the estimate. Red curve show the combined approach and the blue curve show the separate approach.

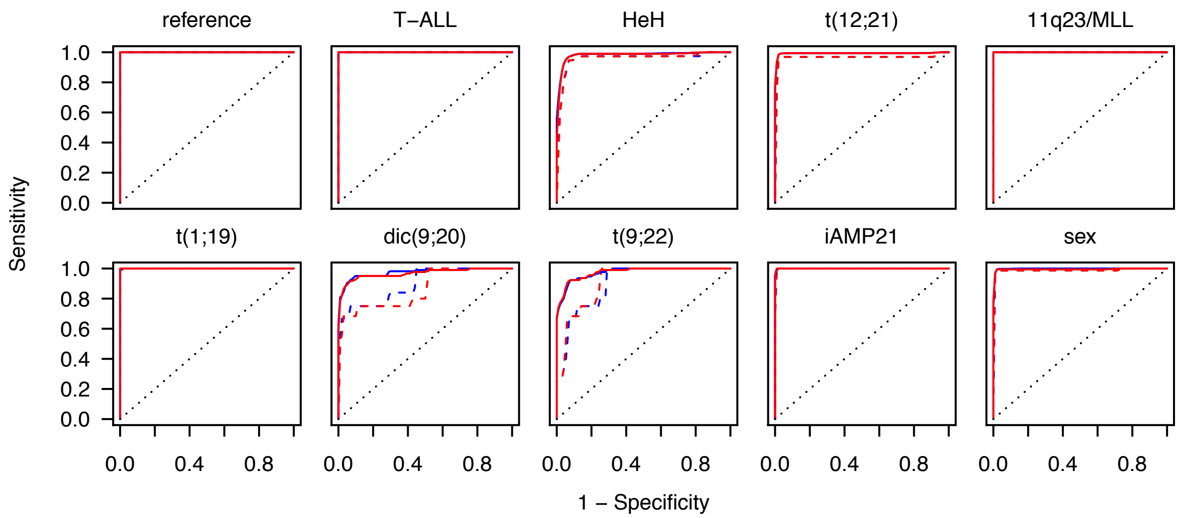

Supplementary Figure S5: *Heatmap of original and newly classified T-ALL samples.*

The patient samples are shown in columns and CpG sites are show in rows. In the heatmap, blue corresponds to low methylation and red to high methylation. Samples denoted with * are newly classified.

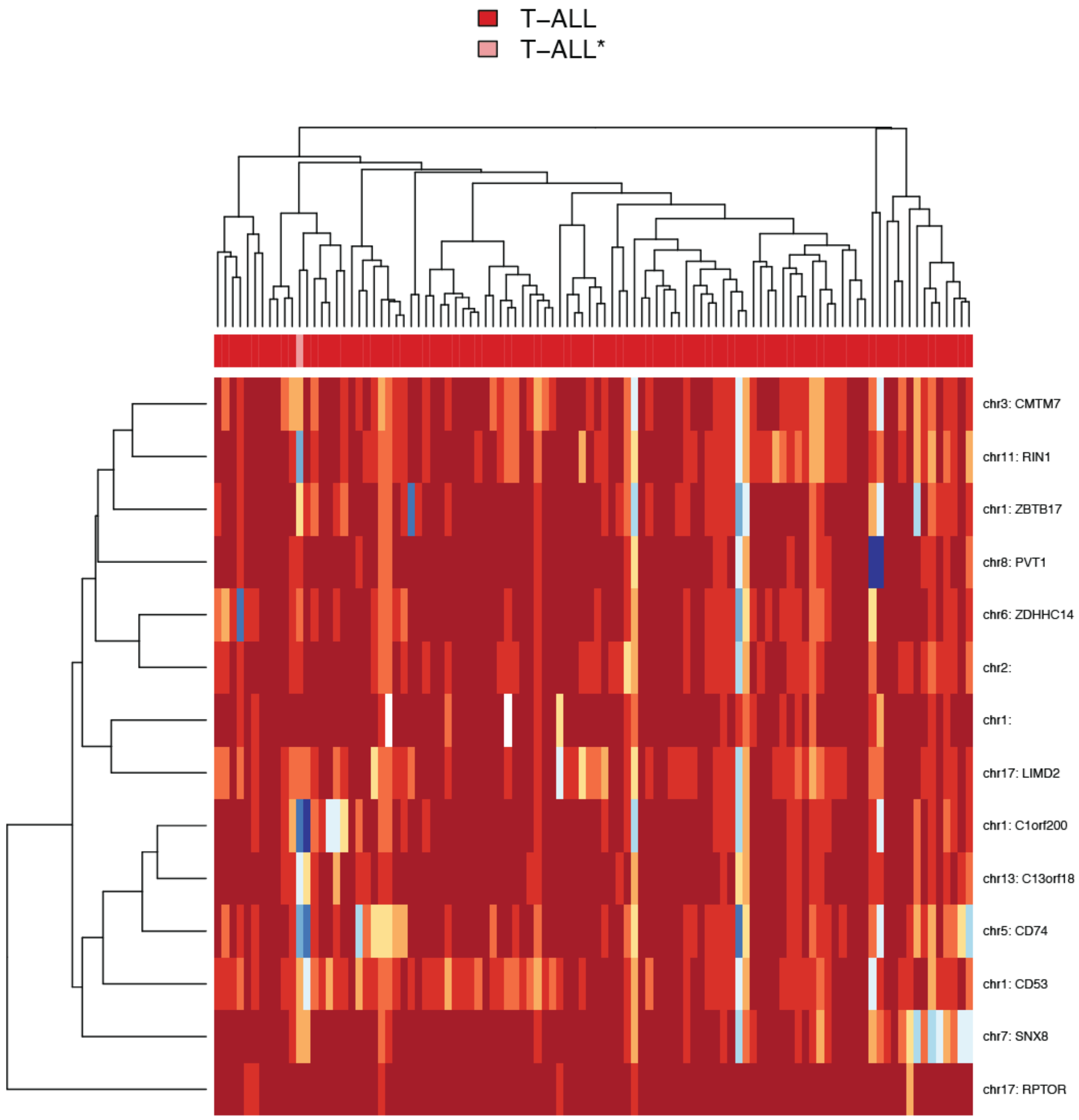

Supplementary Figure S6: *Heatmap of original and newly classified HeH samples.*

The patient samples are shown in columns and CpG sites are show in rows. In the heatmap, blue corresponds to low methylation and red to high methylation. Samples analyzed for fusion genes with RNA-sequencing are shown at the bottom of the figure. Samples denoted with * are newly classified.

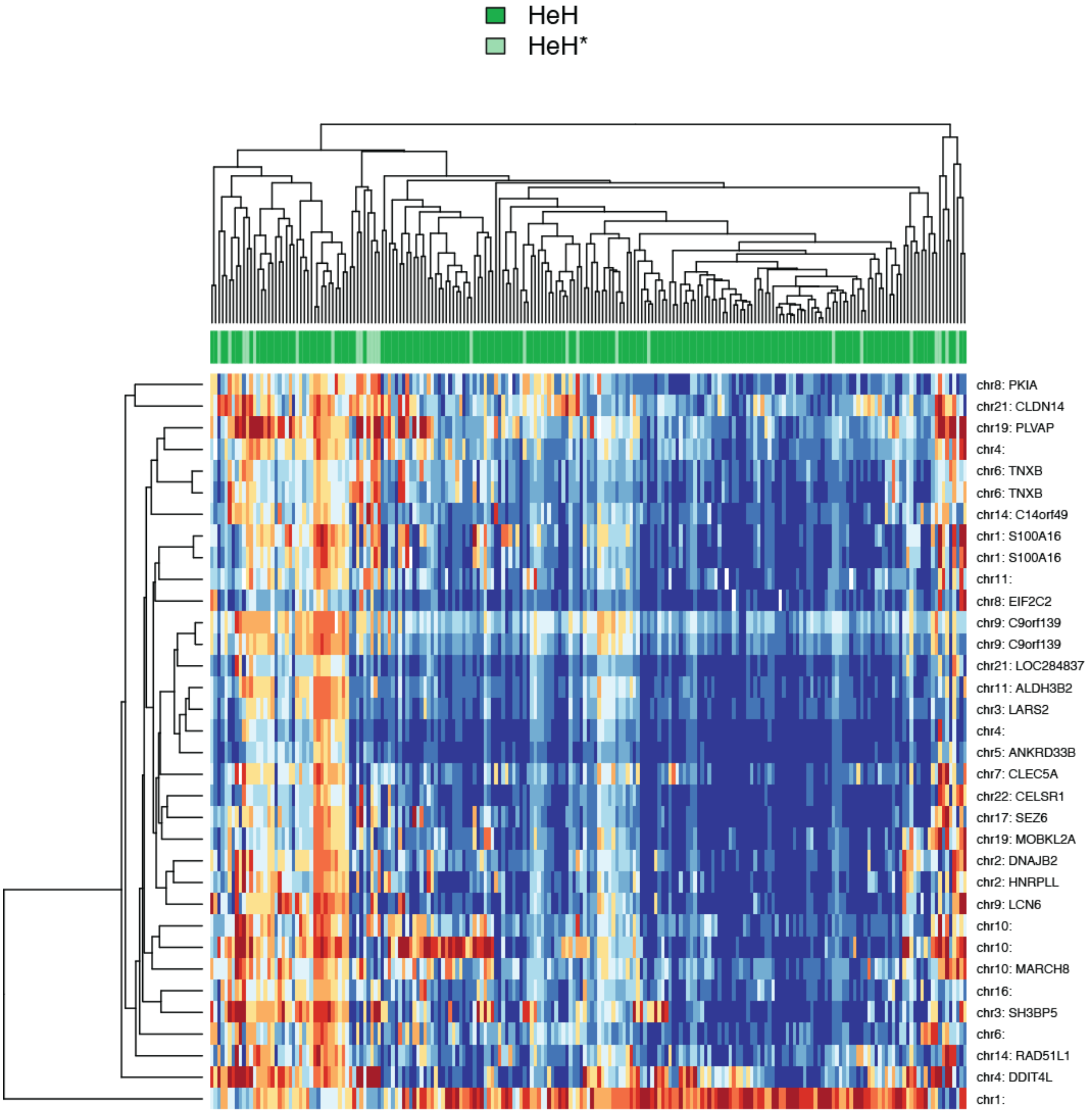

Supplementary Figure S7: *Heatmap of original and newly classified t(12;21) samples.*

The patient samples are shown in columns and CpG sites are show in rows. In the heatmap, blue corresponds to low methylation and red to high methylation. Samples re-analyzed for ETV6/RUNX1 by RT-PCR or analyzed for fusion genes with RNA-sequencing are labeled at the bottom of the heatmap. Samples denoted with * are newly classified.

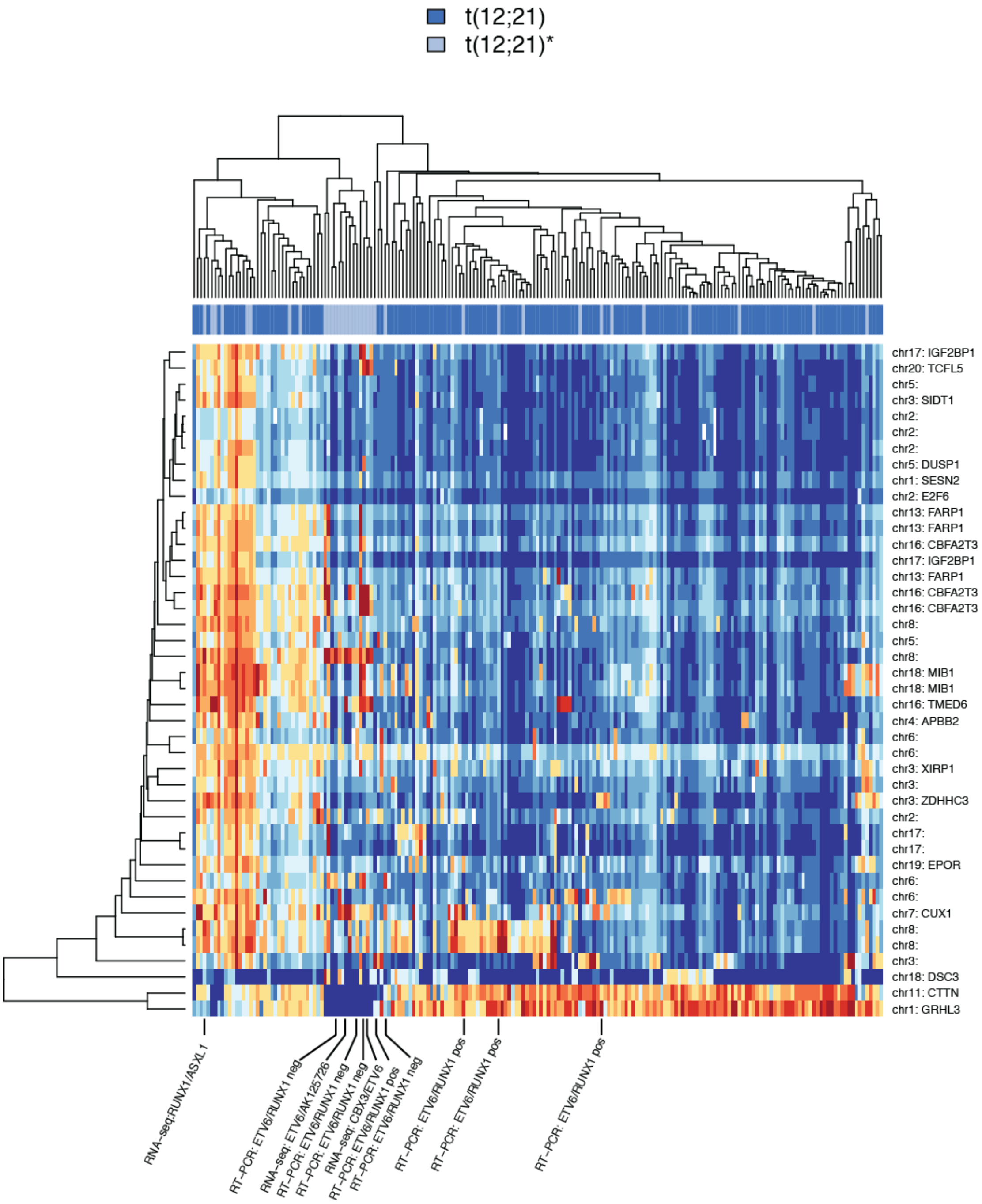

Supplementary Figure S8: *Heatmap of original and newly classified 11q23/MLL samples.*

The patient samples are shown in columns and CpG sites are show in rows. In the heatmap, blue corresponds to low methylation and red to high methylation. Samples denoted with * are newly classified.

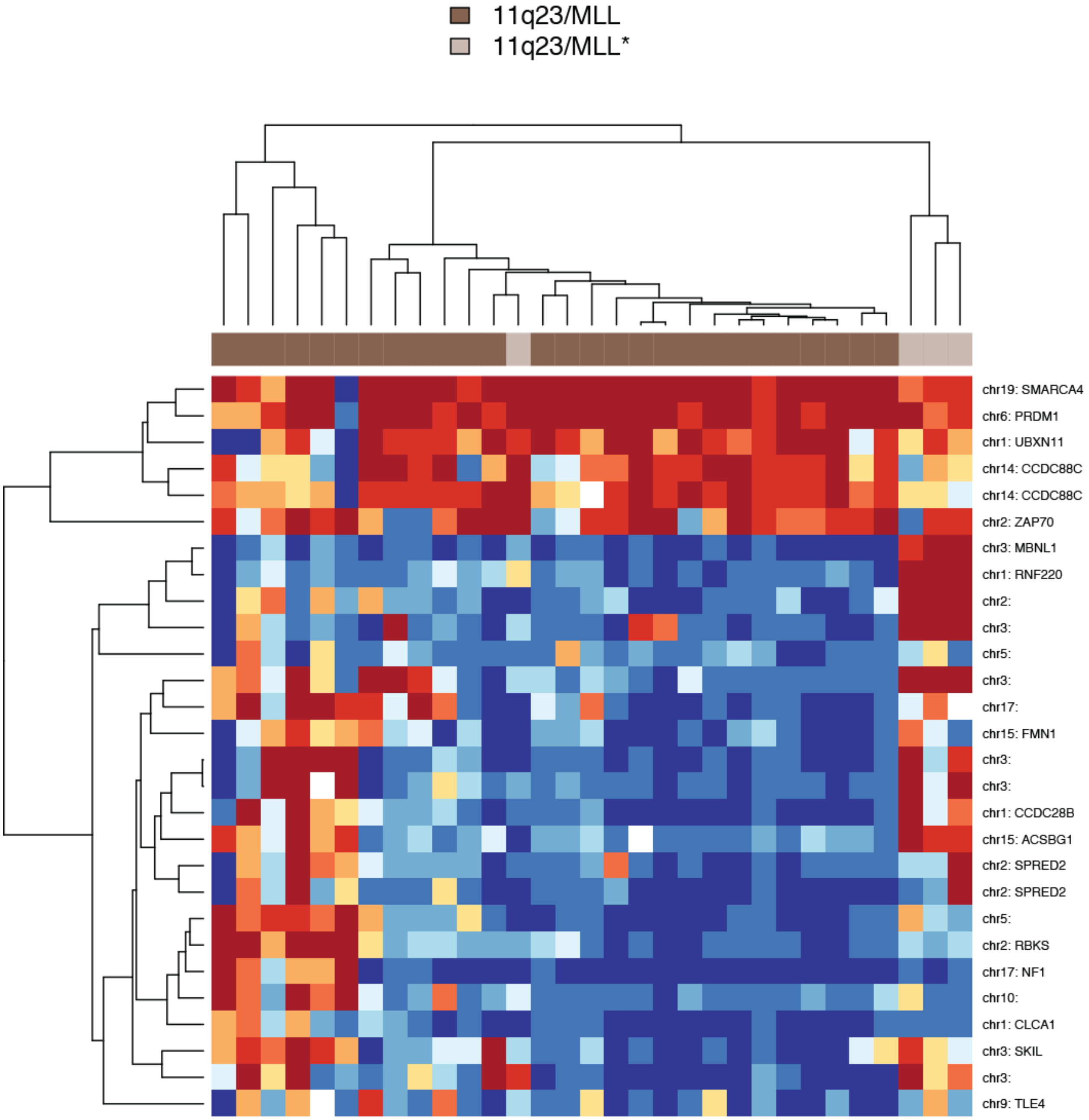

Supplementary Figure S9: *Heatmap of original and newly classified dic(9;20) samples.*

The patient samples are shown in columns and CpG sites are show in rows. In the heatmap, blue corresponds to low methylation and red to high methylation. Samples analyzed for fusion genes with RNA-sequencing are indicated below the heatmap. Samples denoted with * are newly classified.

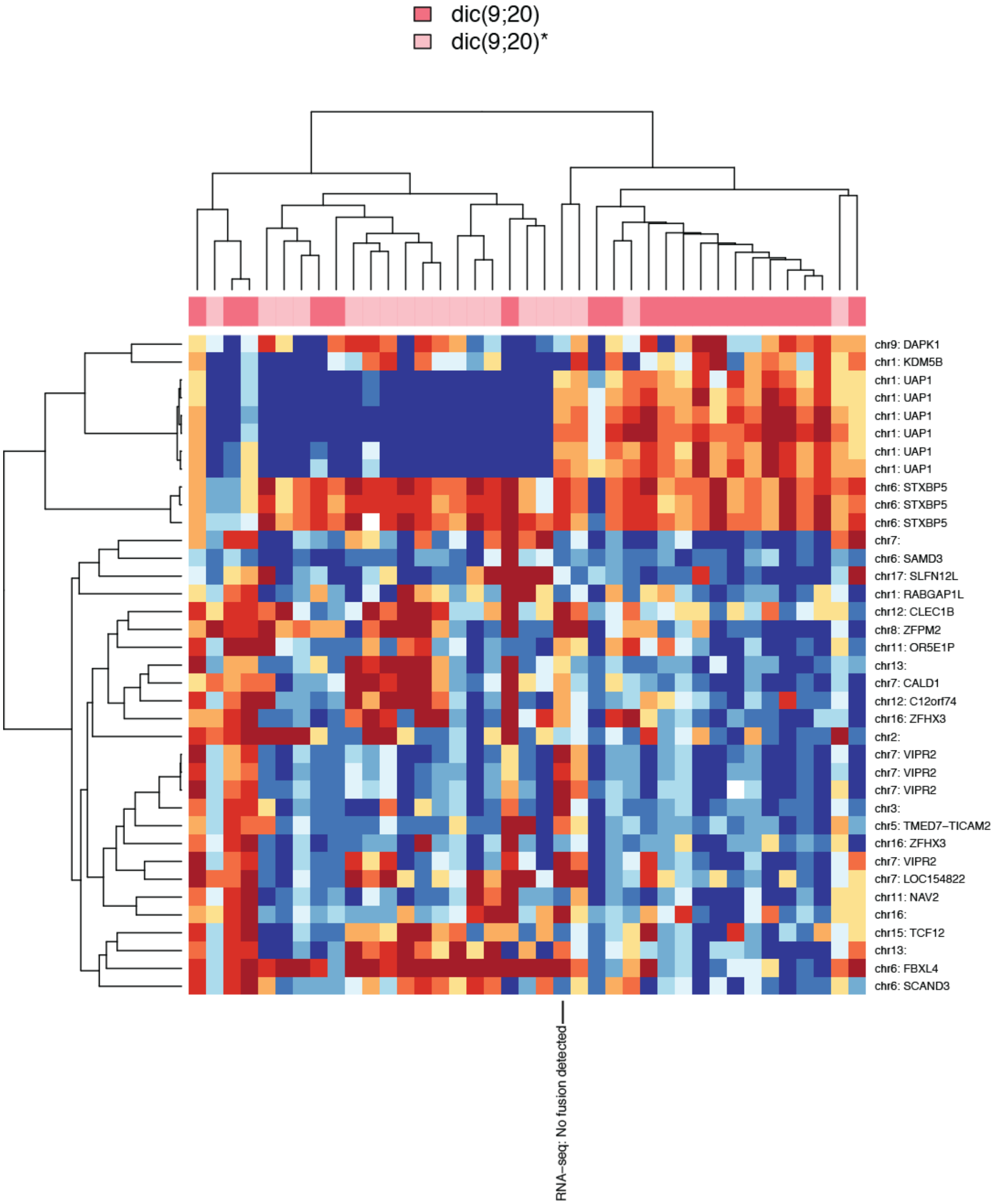

Supplementary Figure S10: *Heatmap of original and newly classified t(1;19) samples.*

The patient samples are shown in columns and CpG sites are show in rows. In the heatmap, blue corresponds to low methylation and red to high methylation. Samples analyzed by RNA-sequencing are indicated below the heatmap. Samples denoted with * are newly classified.

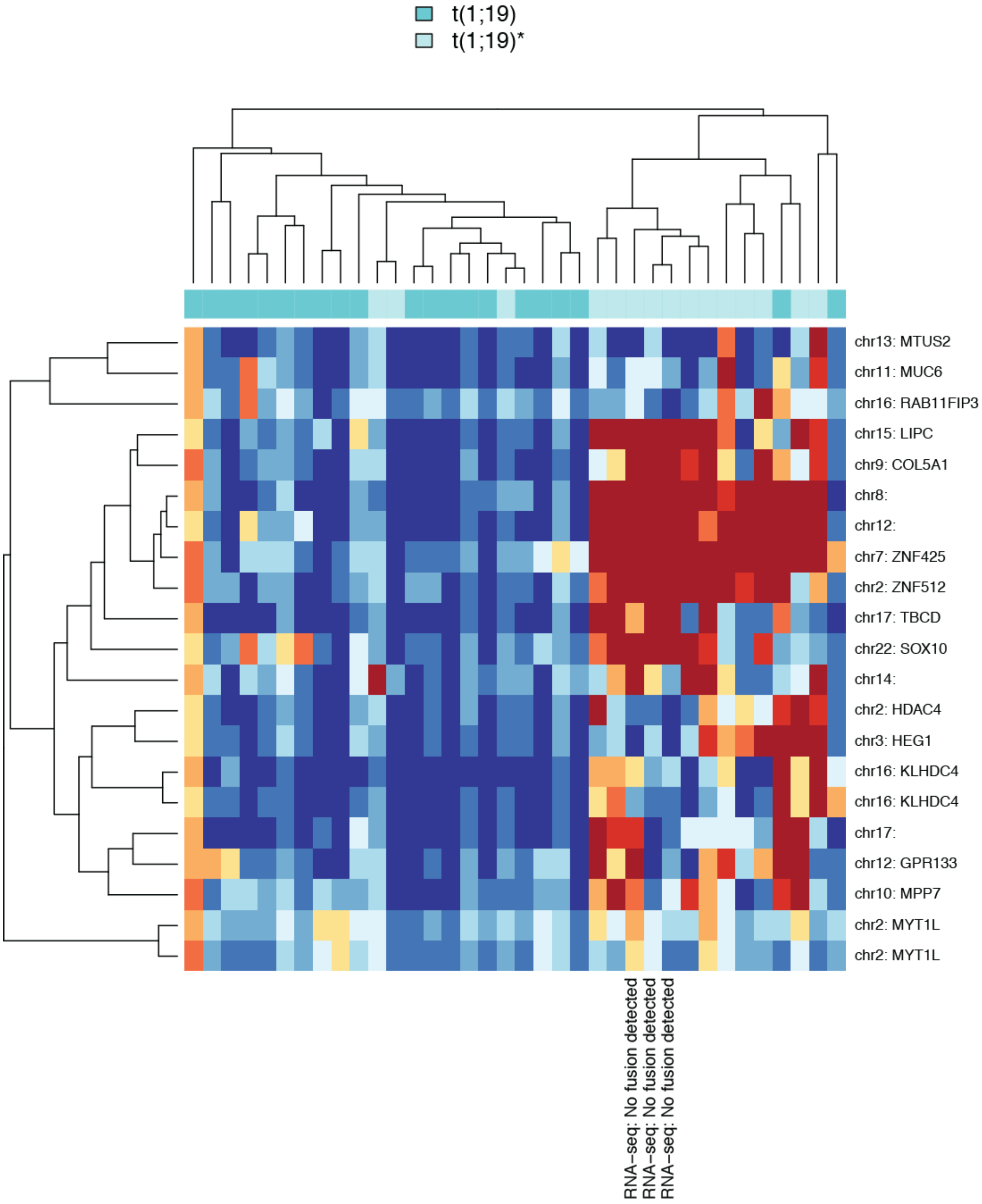

Supplementary Figure S11: *Heatmap of original and newly classified t(9;22) samples.*

The patient samples are shown in columns and CpG sites are show in rows. In the heatmap, blue corresponds to low methylation and red to high methylation. Samples denoted with * are newly classified.

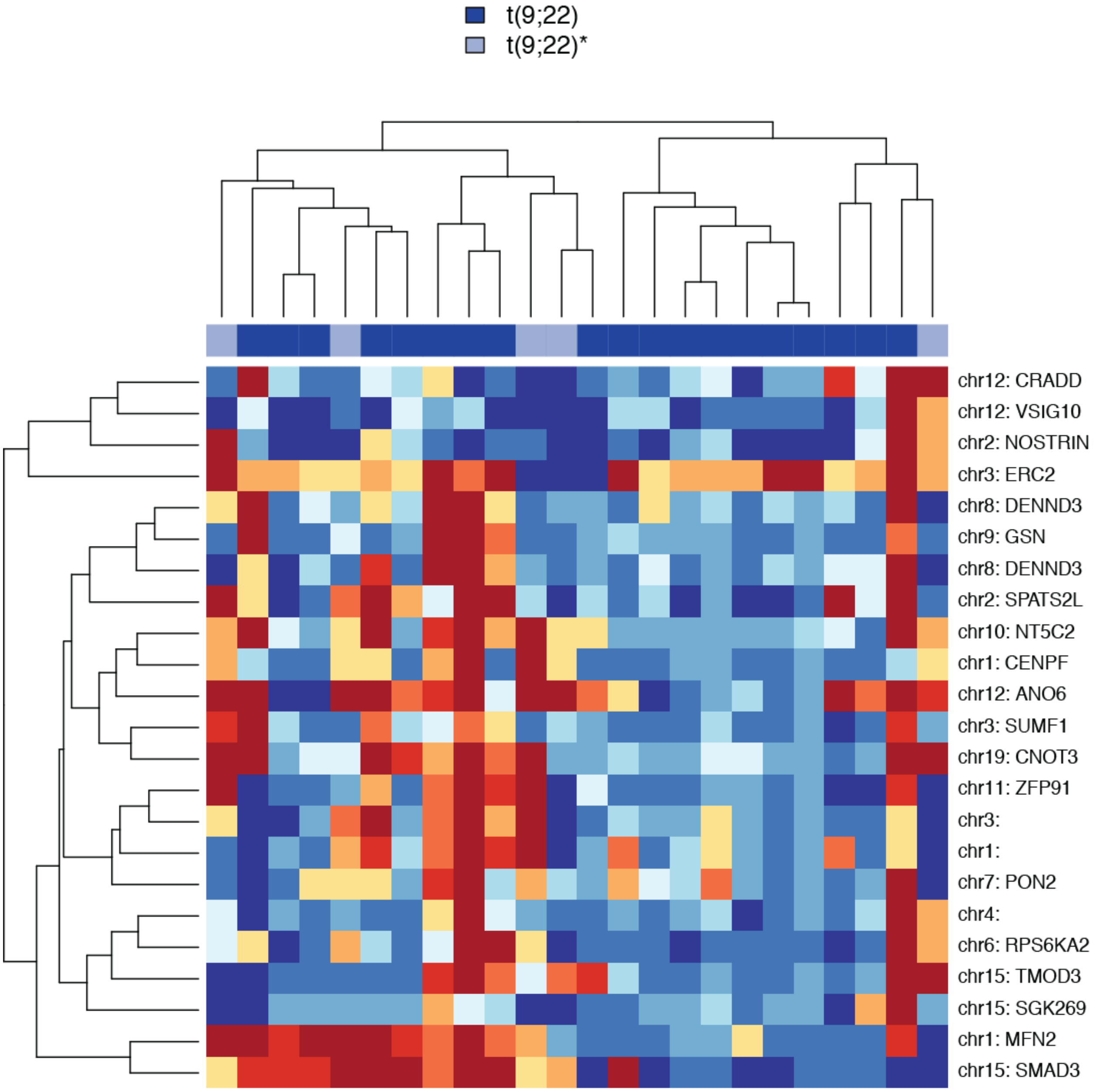

Supplementary Figure S12: *Heatmap of original and newly classified iAMP21 samples.*

The patient samples are shown in columns and CpG sites are show in rows. In the heatmap, blue corresponds to low methylation and red to high methylation. Samples denoted with * are newly classified.

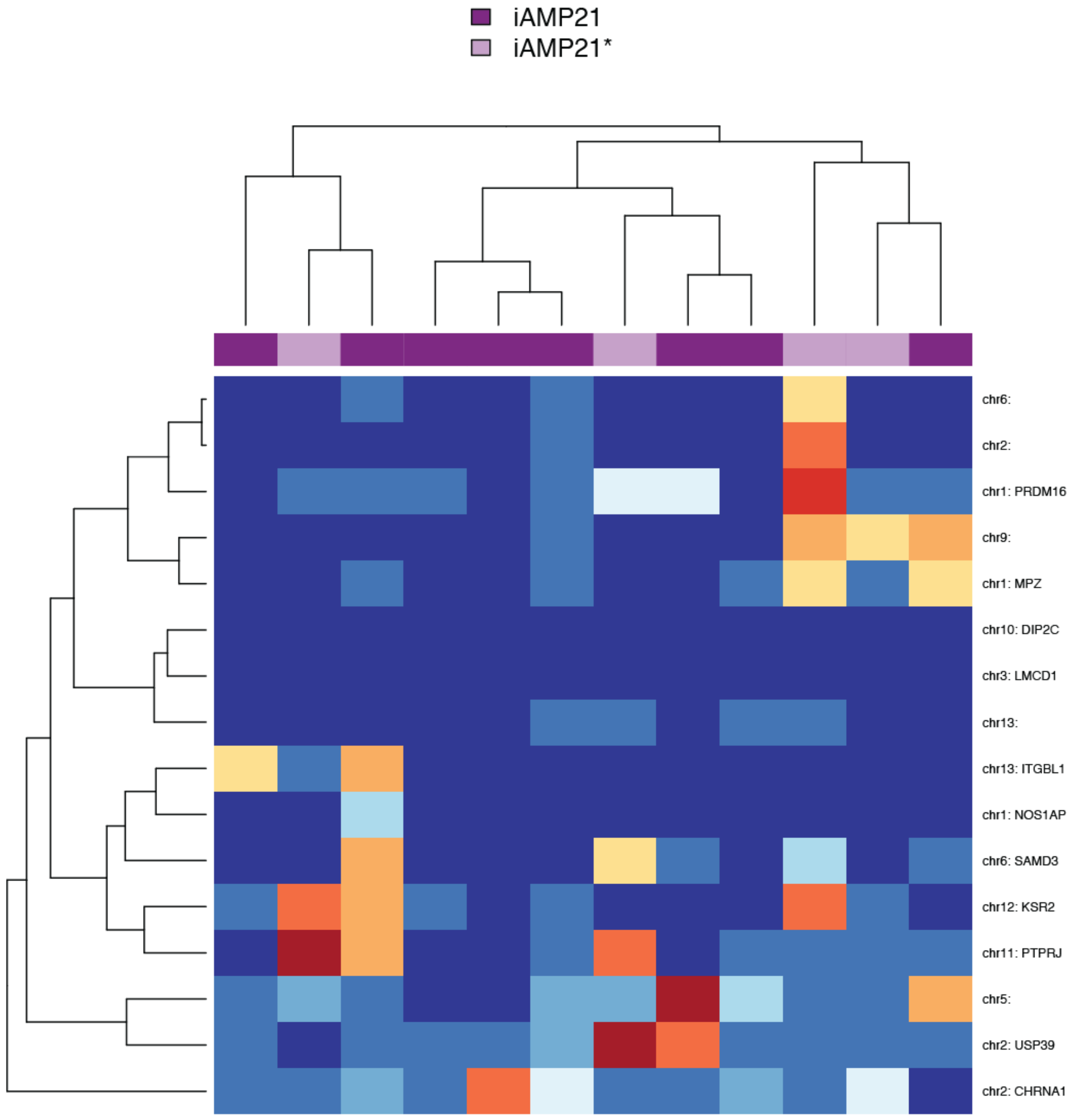

Supplementary Figure S13: *Heatmap of samples newly classified in more than one subtype (multi-class) or without any subtype classified (non-class).*

The patient samples are shown in columns and CpG sites are show in rows. In the heatmap, blue corresponds to low methylation and red to high methylation. Patients analyzed by RNA-sequencing are indicated at the bottom of the heatmap.

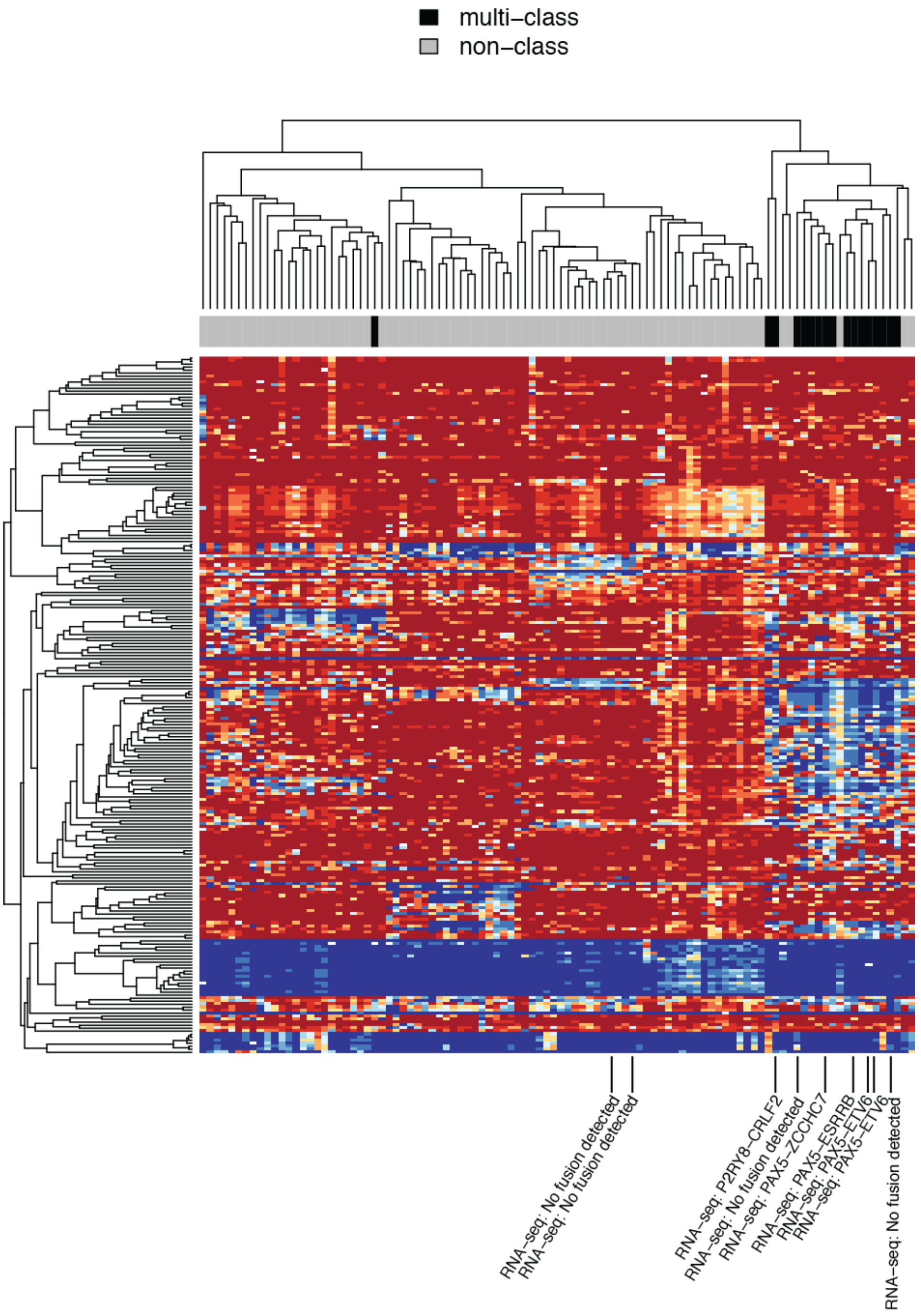

Supplementary Figure S14: *Whole genome copy number heat map in HeH (n=189) and newly classified HeH* (n=25) cases.*

Each row is a sample, grouped by methylation subtyping and annotated with original cytogenetic grouping and methylation classifier scores (white to blue gradient, 0-1). Loss and gain of genomic material is indicated in blue and red respectively.

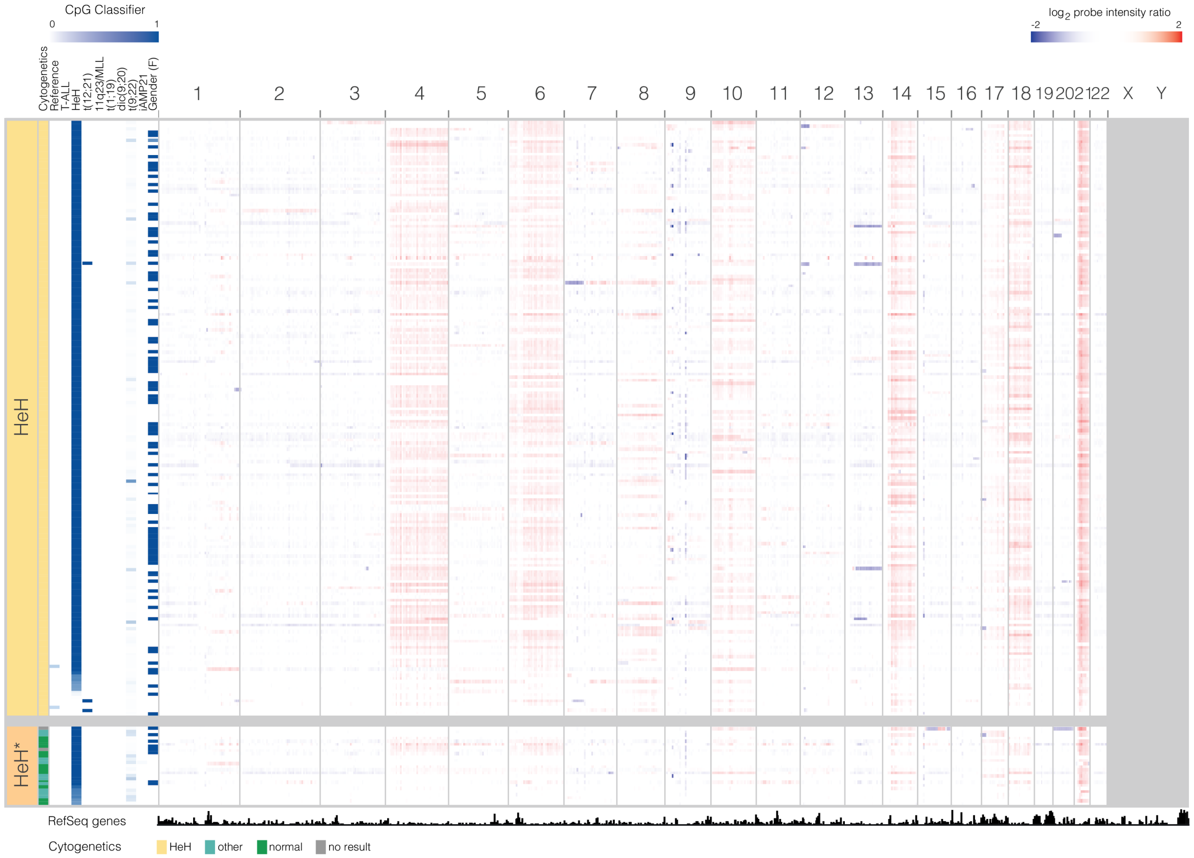

Supplementary Figure S15: *Whole genome copy number heat map in iAMP21 (n=8) and newly classified iAMP21* (n=4) cases*.

Each row is a sample, grouped by methylation subtyping and annotated with original cytogenetic grouping and methylation classifier scores (white to blue gradient, 0-1). Loss and gain of genomic material is indicated in blue and red respectively.

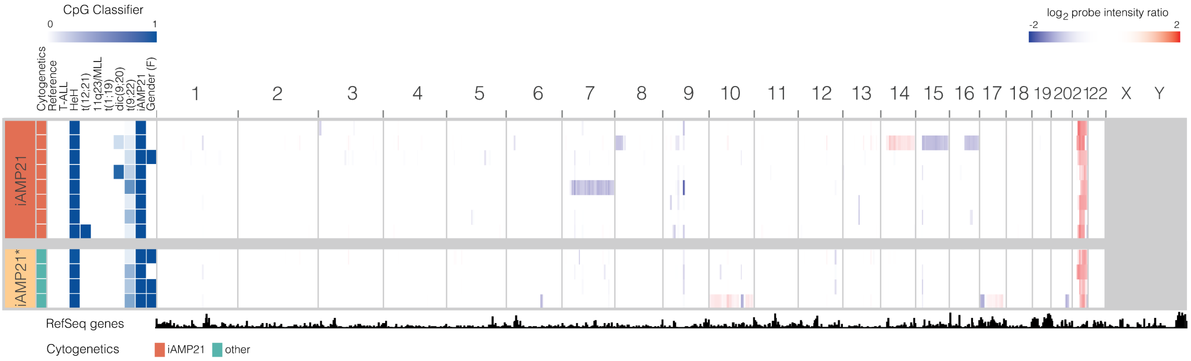

Supplementary Figure S16: *Genome-wide copy number heat map in dic(9;20) (n=20), newly classified dic(9;20)* (n=19), and multi-class (n=17) cases*.

Each row is a sample, grouped by methylation subtyping and annotated with original cytogenetic grouping and methylation classifier scores (white to blue gradient, 0-1). Loss and gain of genomic material is indicated in blue and red respectively. Whole genome copy number heat map in dic(9;20) (n=20), dic(9;20)* (n=19) and Multi-class (n=17) cases. Each row is a sample, grouped by methylation subtyping and annotated with cytogenetic grouping at diagnosis and methylation-based classifier scores. Loss and gain of genomic material is indicated in blue and red respectively. Sample key:*del(9p), del(20q); ** No fusion gene detected; ***PAX5-ETV6; ****P2RY8-CRLF2; *****PAX5-ESRRB; ******PAX5-ZCCHC7.

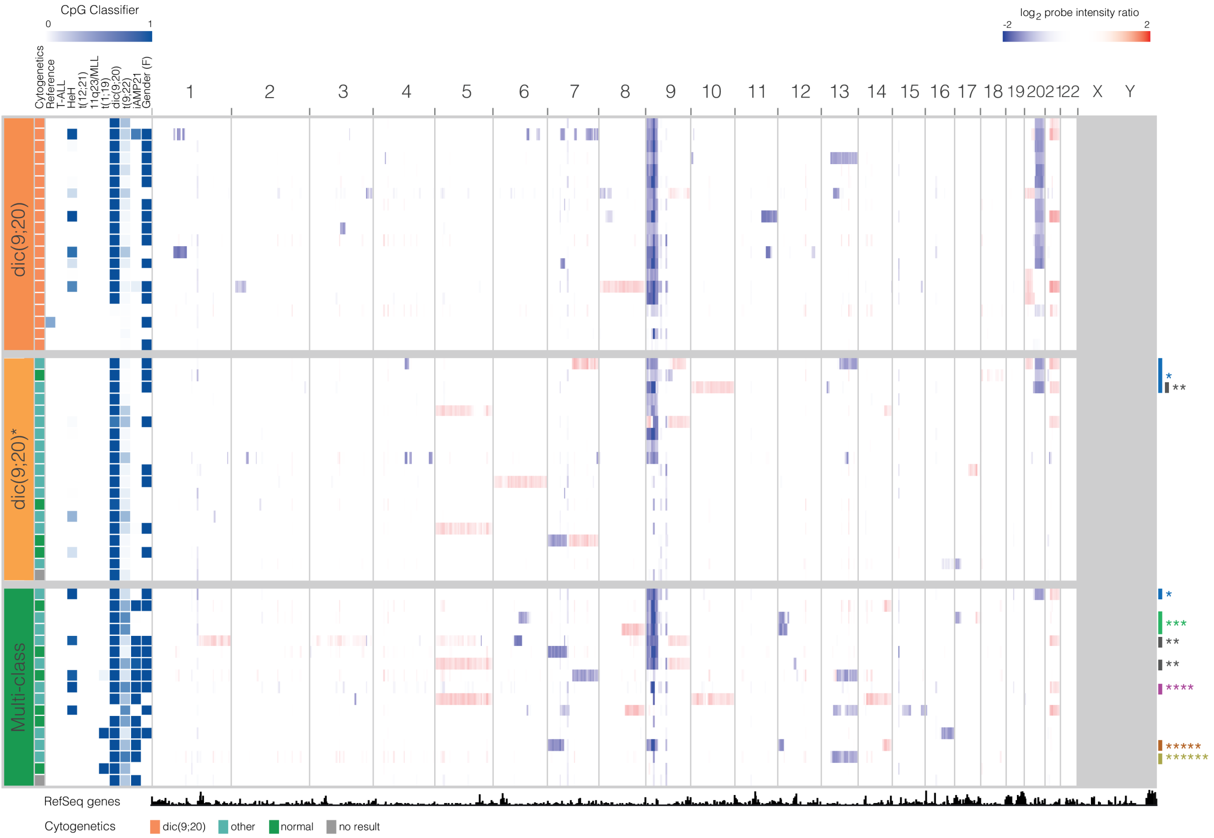

Supplementary Figure S17: *Genome-wide copy number heat map in t(1;19) (n=21) and newly classified t(1;19)* (n=15) cases.*

Each row is a sample, grouped by methylation subtyping and annotated with original cytogenetic grouping and methylation classifier scores (white to blue gradient, 0-1). Loss and gain of genomic material is indicated in blue and red respectively. *No fusion gene detected.

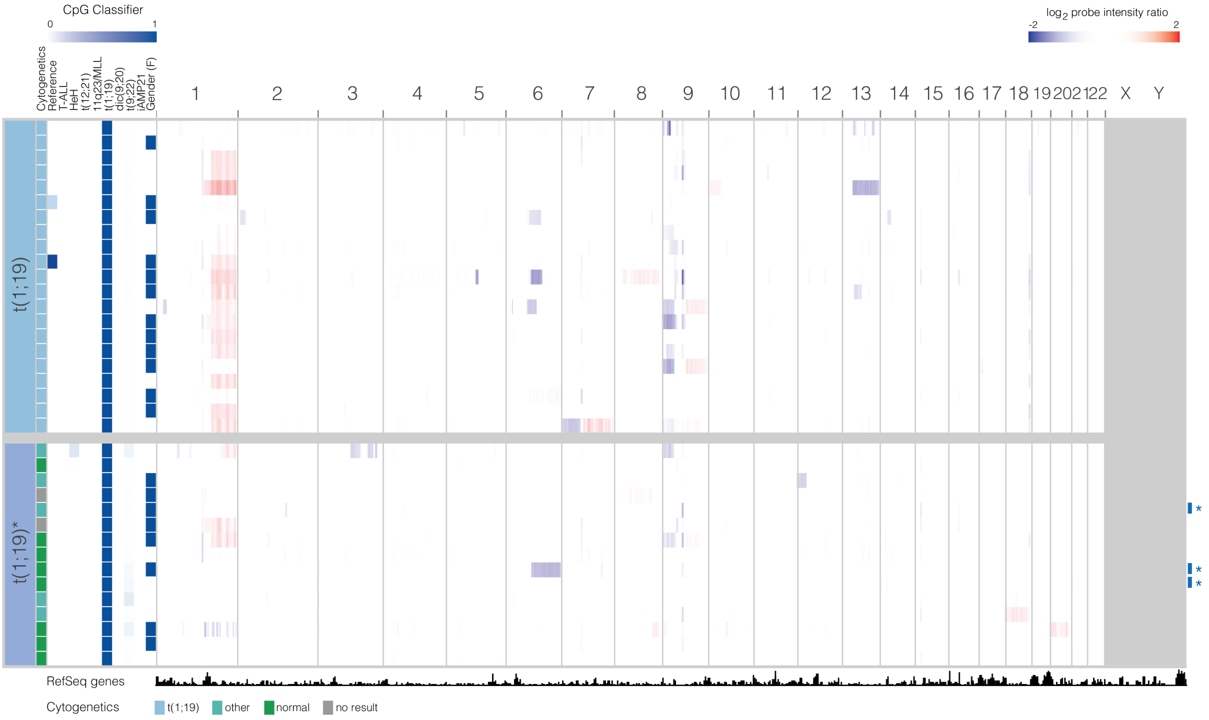

Supplementary Figure S18: *Genome-wide copy number heat map in t(9;22) (n=19) and newly classified t(9;22)* (n=5) cases.*

Each row is a sample, grouped by methylation subtyping and annotated with original cytogenetic grouping and methylation classifier scores (white to blue gradient, 0-1). Loss and gain of genomic material is indicated in blue and red respectively.

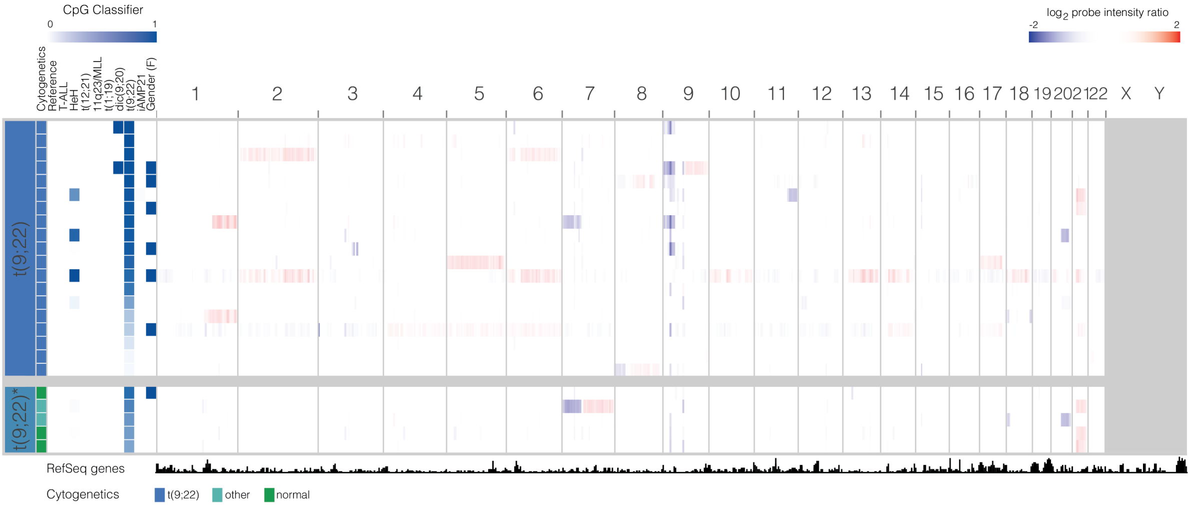

Supplementary Figure S19: *The cumulative incidence of relapse of patients with previously established ALL subtypes and newly classified subtype-like groups treated according to NOPHO protocols.*

a-g) In each panel the cumulative incidence of relapse for patients with previously established ALL subtypes are shown as a colored line and the newly classified subtype-like group is indicated by the black curve. h) Cumulative incidence of relapse of ALL patients that were not assigned to any subtype group by the classifier (gray) and patients that were assigned to multiple subtypes (black). The Gray's test p-value for difference between the two groups is indicated at the bottom of each panel if the minimum number of patients in each group exceeded 10. Competing events are considered censoring.

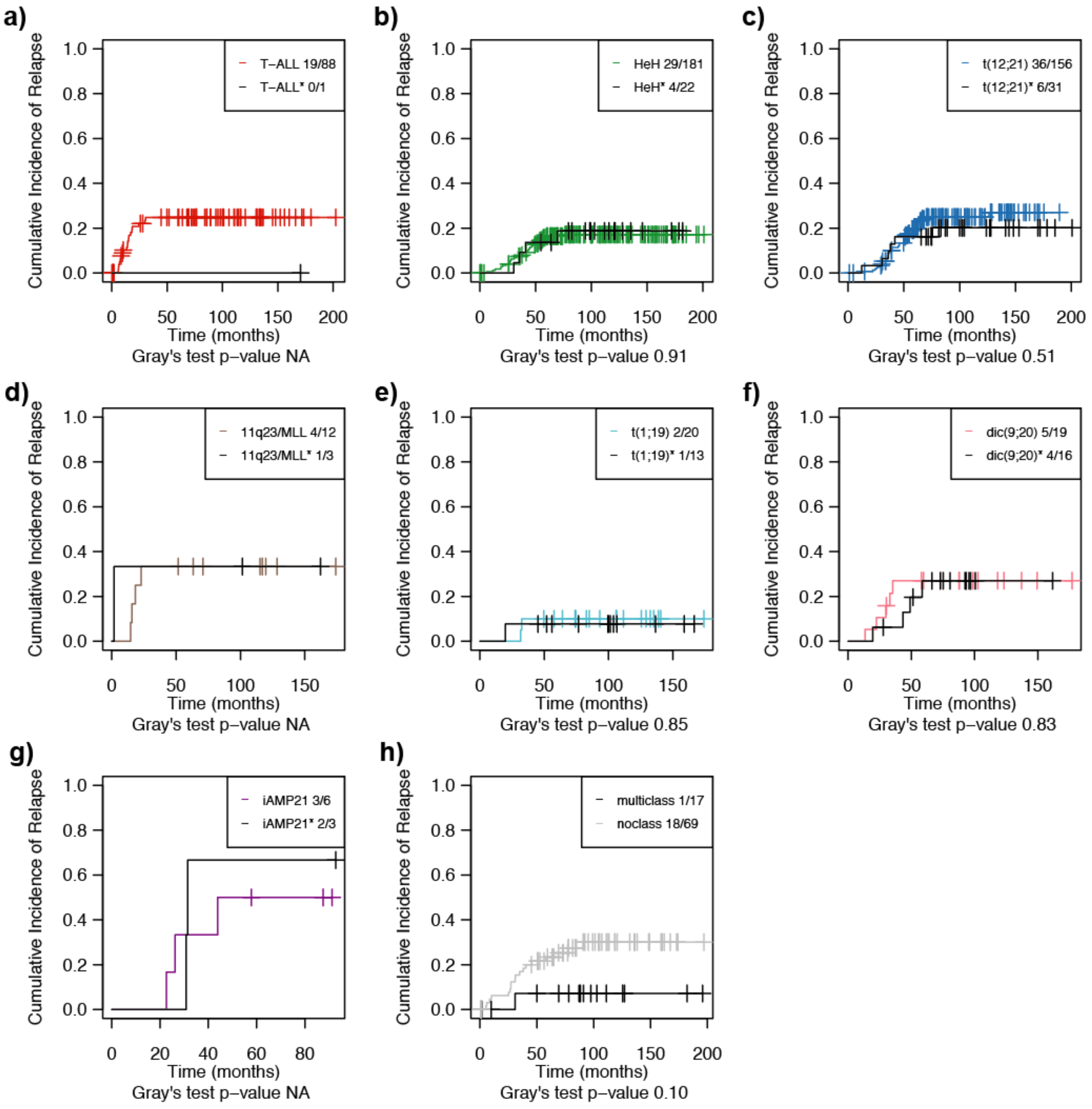

Supplementary Figure S20: *The cumulative incidence of relapse of patients with previously established ALL subtypes treated according to specialized treatment protocols and the newly classified subtype-like patients.*

a) The cumulative incidence of relapse for patients with previously established 11q23/MLL subtype treated according to the NOPHO-infant99 or NOPHO-infant06 protocols are shown in brown and newly classified 11q23/MLL-like group is indicated by the black curve. b) The cumulative incidence of relapse for patients with previously established t(9;22) subtype treated according to the Ph+/NOPHO-ALL or EsPhALL protocols are shown in blue and newly classified t(9;22)-like group is indicated by the black curve. Information about the treatment protocols for the 11q23/MLL-like and t(9;22)-like patients can be found in Table 5. Competing events are considered censoring.

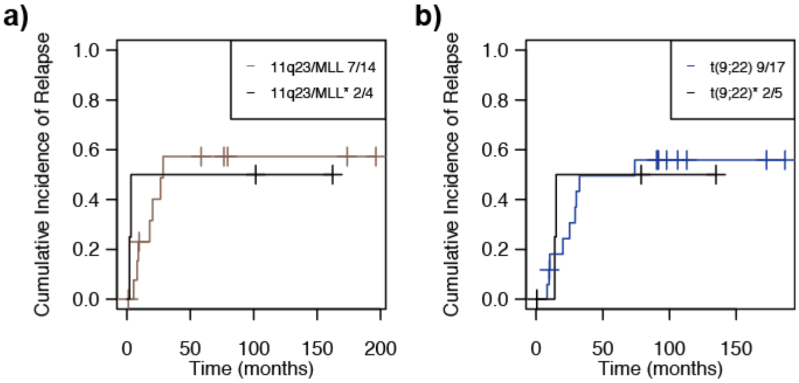

Supplement: Additional file 1: — Supplementary methods and supplementary Figures S1–S20. [file 13148_2014_39_MOESM1_ESM.pdf]
